# Supplementary material for: Nef-Mediated CD3-TCR Downmodulation Dampens Acute Inflammation and Promotes SIV Immune Evasion
Source: Cell Rep. Author manuscript; Available in PMC 2020 Mar 3. (PMC7052273; doi:10.1016/j.celrep.2020.01.069)
Supplement: 1 [file NIHMS1563620-supplement-1.pdf]

Cell Reports, Volume *30*

Supplemental Information

Nef-Mediated CD3-TCR Downmodulation Dampens Acute Inflammation and Promotes SIV Immune Evasion

Simone Joas, Ulrike Sauermann, Berit Roshani, Antonina Klippert, Maria Daskalaki, Kerstin Mätz-Rensing, Nicole Stolte-Leeb, Anke Heigele, Gregory K. Tharp, Prachi Mehrotra Gupta, Sydney Nelson, Steven Bosinger, Laura Parodi, Luis Giavedoni, Guido Silvestri, Daniel Sauter, Christiane Stahl-Hennig, and Frank Kirchhoff

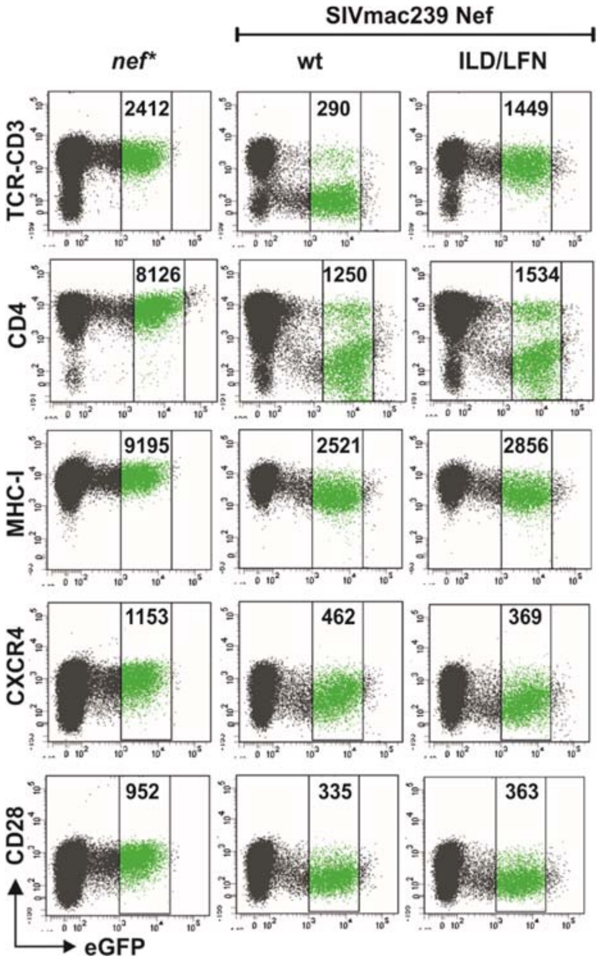

Figure S1 (related to Figure 1). Modulation of CD3, CD4, MHC-I, CXCR4 and CD28 by SIV$_{mac239}$ wt and CD3ko (ILD/LFN) Nef proteins. Human PBMCs were stimulated with PHA for three days and subsequently transduced with VSV-G-pseudotyped HIV-1 NL4-3 IRES-eGFP constructs containing a defective *nef* gene (*nef**) or the wt or mutant SIV$_{mac239}$ *nef* alleles. Surface expression was analyzed by flow cytometric analysis 2 days later.

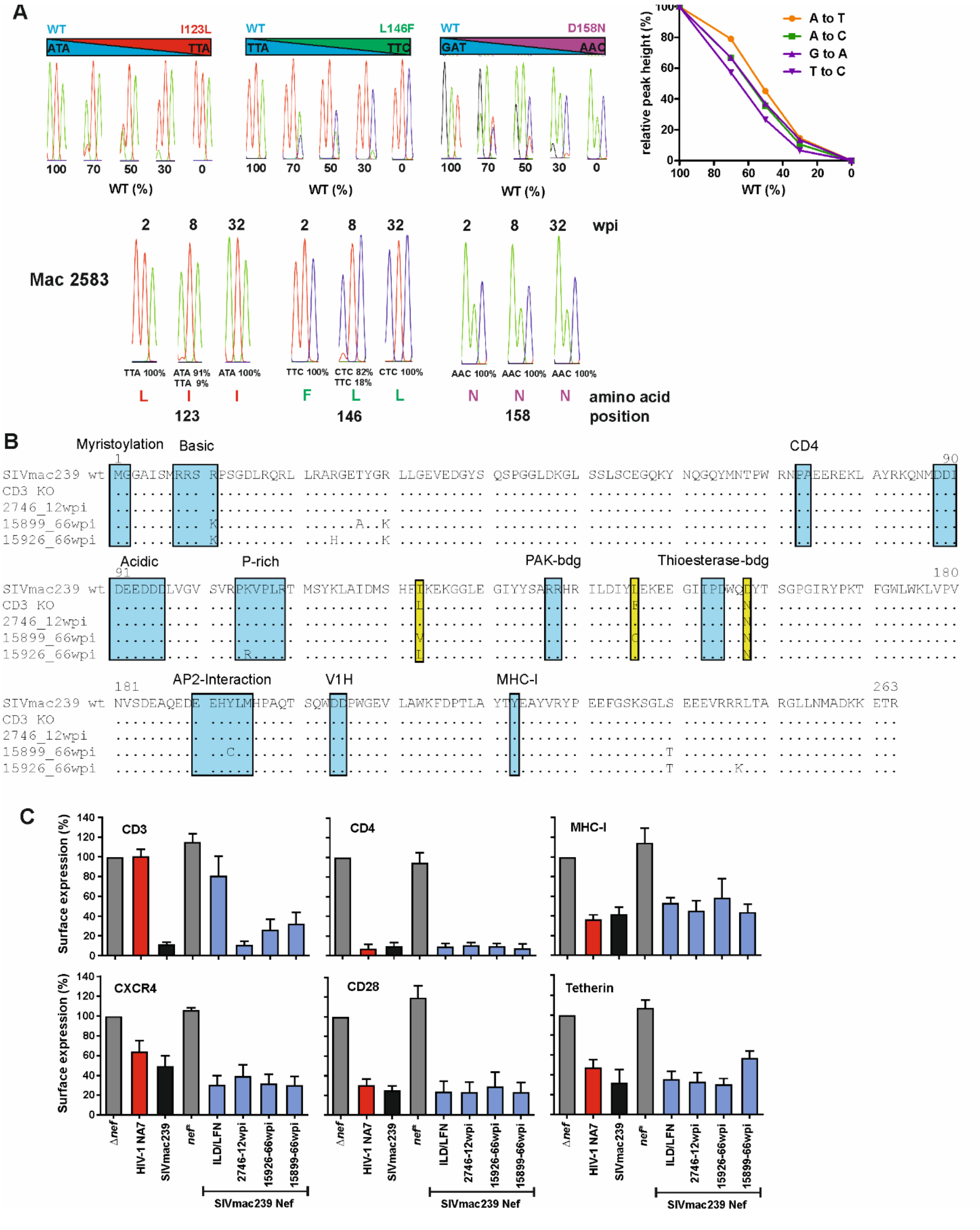

A
WT I123L ATA TTA
WT L146F TTA TTC
WT D158N GAT AAC
100 70 50 30 0
WT (%)
relative peak height (%)
A to T
A to C
G to A
T to C
Mac 2583
2 8 32 wpi
TTA 100% ATA 91% TTA 9% ATA 100%
TTC 100% CTC 82% TTC 18% CTC 100%
AAC 100% AAC 100% AAC 100%
L I I F L L N N N
123 146 158 amino acid position
B
Myristoylation Basic CD4
SIVmac239 wt
CD3 KO
2746_12wpi
15899_66wpi
15926_66wpi
Acidic P-rich PAK-bdg Thioesterase-bdg
AP2-Interaction V1H MHC-I
C
CD3 CD4 MHC-I CXCR4 CD28 Tetherin
Surface expression (%)
Δnef
HIV-1 NA7
SIVmac239
nef*
ILD/LFN
2746-12wpi
15926-66wpi
15899-66wpi
SIVmac239 Nef

Figure S2 (related to Figure 2). Detection and functional analysis of reversions detected in rhesus macaques infected with the CD3ko-Nef SIV_{mac239} construct. (**A**) Quantitative detection of reversions in the CD3ko-Nef. Upper, PCR amplification products of wt and *nef* mutant SIV_{mac239} constructs were mixed at the indicated molar ratios and sequenced as described in the methods section. The right panel shows the percentage of peak fluorescence for wt nucleotides compared with the total fluorescence. Lower, RNA was isolated from plasma of animal 2583 at the indicated weeks post-infection, the *nef* coding region amplified by RT-PCR and sequenced directly. The percentage of reversions was calculated from the standard curves shown above. (**B**) Alignment of the wild-type and CD3ko-Nef SIV_{mac239} Nef amino sequences with those detected in animals 2746, 15899 and 15296 at the indicated weeks post-infection. Dots represent amino acid identity; known functional domains are indicated. (**C**) Human PBMCs were transduced with VSV-G-pseudotyped HIV-1 NL4-3 IRES-eGFP constructs expressing the indicated *nef* alleles and assayed for surface expression of CD3, CD4, MHC-I, CXCR4 and CD28. Shown are average values ± SD obtained for three different donors. To measure the effect on tetherin, HEK293T cells were cotransfected with a pCG vector expressing macaque tetherin and an NL4-3 proviral construct expressing the indicated *nef* alleles. Two days later, cell surface expression of tetherin was determined by FACS.

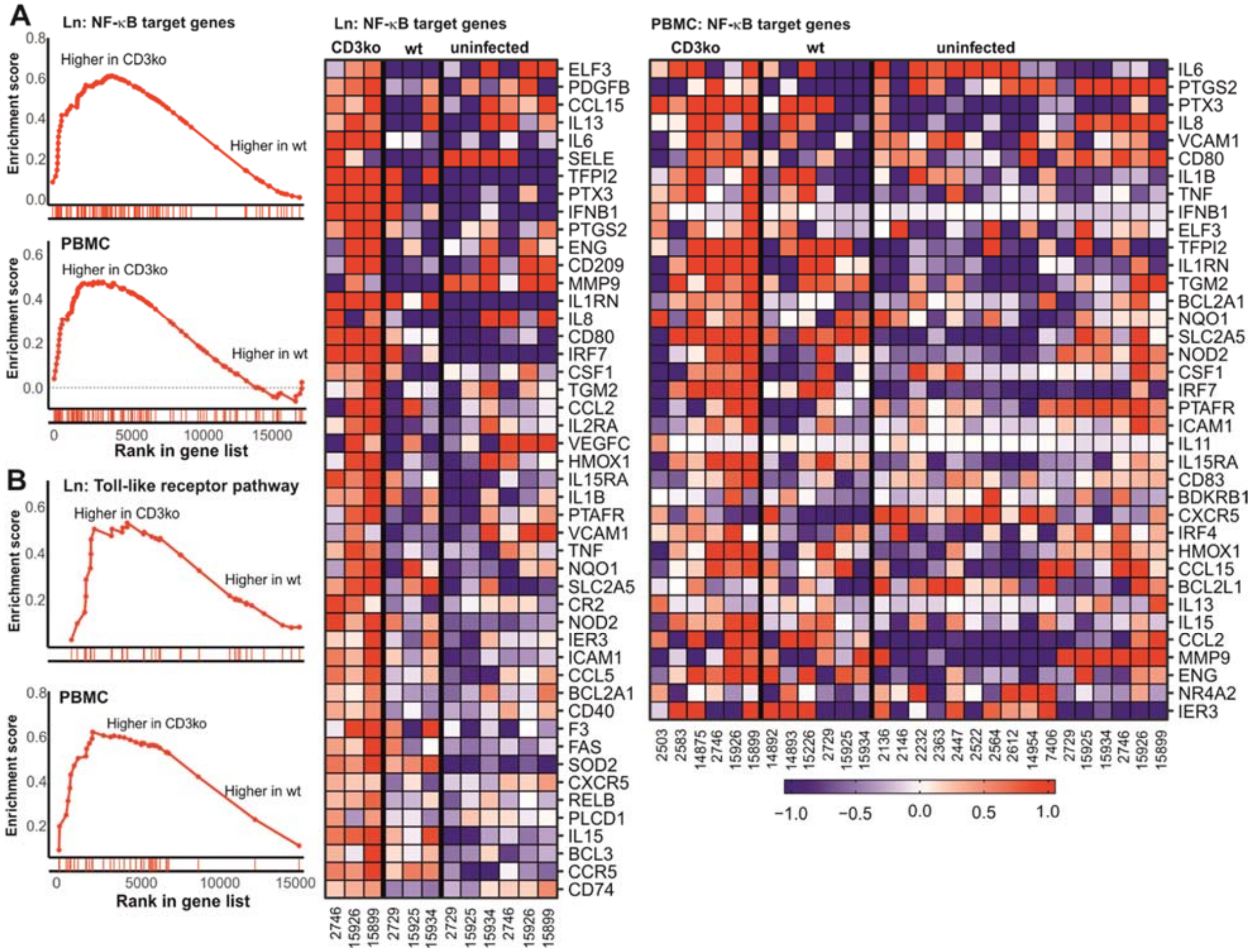

Figure S3 (related to Figure 3). Lack of Nef-mediated CD3 downmodulation affects expression of genes involved in NF-κB and TLR signaling. (**A, B**) GSEA plots and heat maps of CD3ko-Nef vs wt in lymph node and PBMC datasets for (A) NF-κB target gene sets and (B) the TLR signaling pathway gene set. The CD3ko-Nef and wt datasets consist of samples collected at 2 wpi from CD3ko-Nef and wt SIV_{mac239} infected animals, respectively. The running enrichment score (y-axis) is indicated for each gene ordered by their rank in the whole data set for that specific comparison (shown by the bars below the x-axis). The right panels show heat maps for the leading-edge genes of the NF-κB target gene set for PBMC and lymph node samples of the CD3ko-Nef and wt at 2 wpi, and uninfected datasets. The color scale is shown at the bottom, with lowest to highest gene expression across all animals represented by the blue to red color gradient

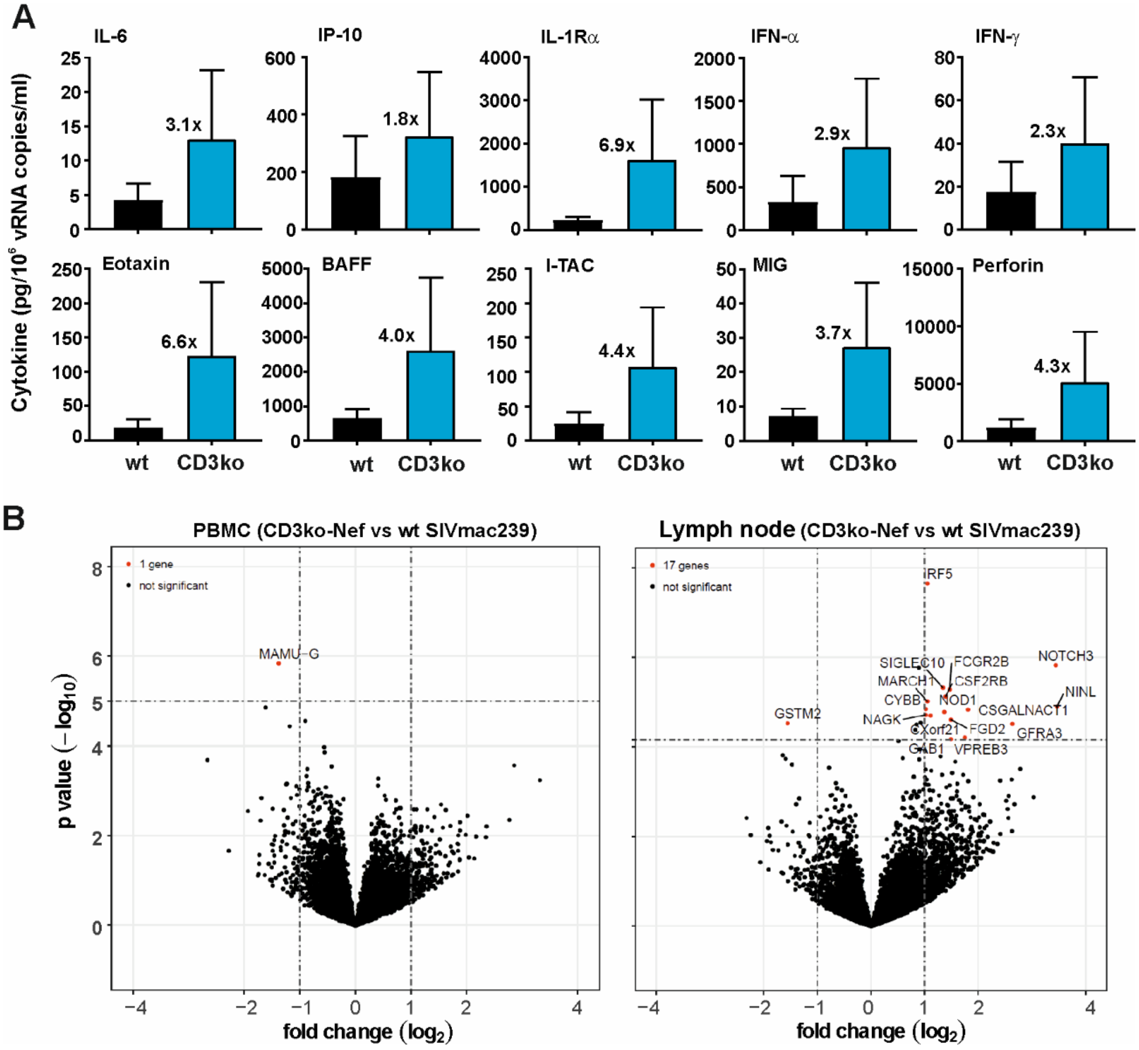

Figure S4 (related to Figure 4). Proinflammatory cytokines and differential gene expression in wt and CD3ko-Nef SIV$_{mac239}$ infection. (**A**) Levels of proinflammatory cytokines in plasma of the 12 rhesus macaques infected with wt or CD3ko-Nef SIV$_{mac239}$ (mean ±SEM) were measured by Luminex. Shown are the quantities of cytokines detected at peak viremia (2 wpi) normalized to the RNA levels of the individual animals. The numbers next to the blue bars indicate n-fold difference. (**B**) Volcano plots depicting the fold-change (CD3ko-Nef vs wt SIV mac239 at 2 wpi) and statistical significance for genes from (left) PBMC and (right) lymph nodes of rhesus macaques. The red dots highlight the differentially expressed genes showing >2-fold change in their expression, with an FDR <0.05.

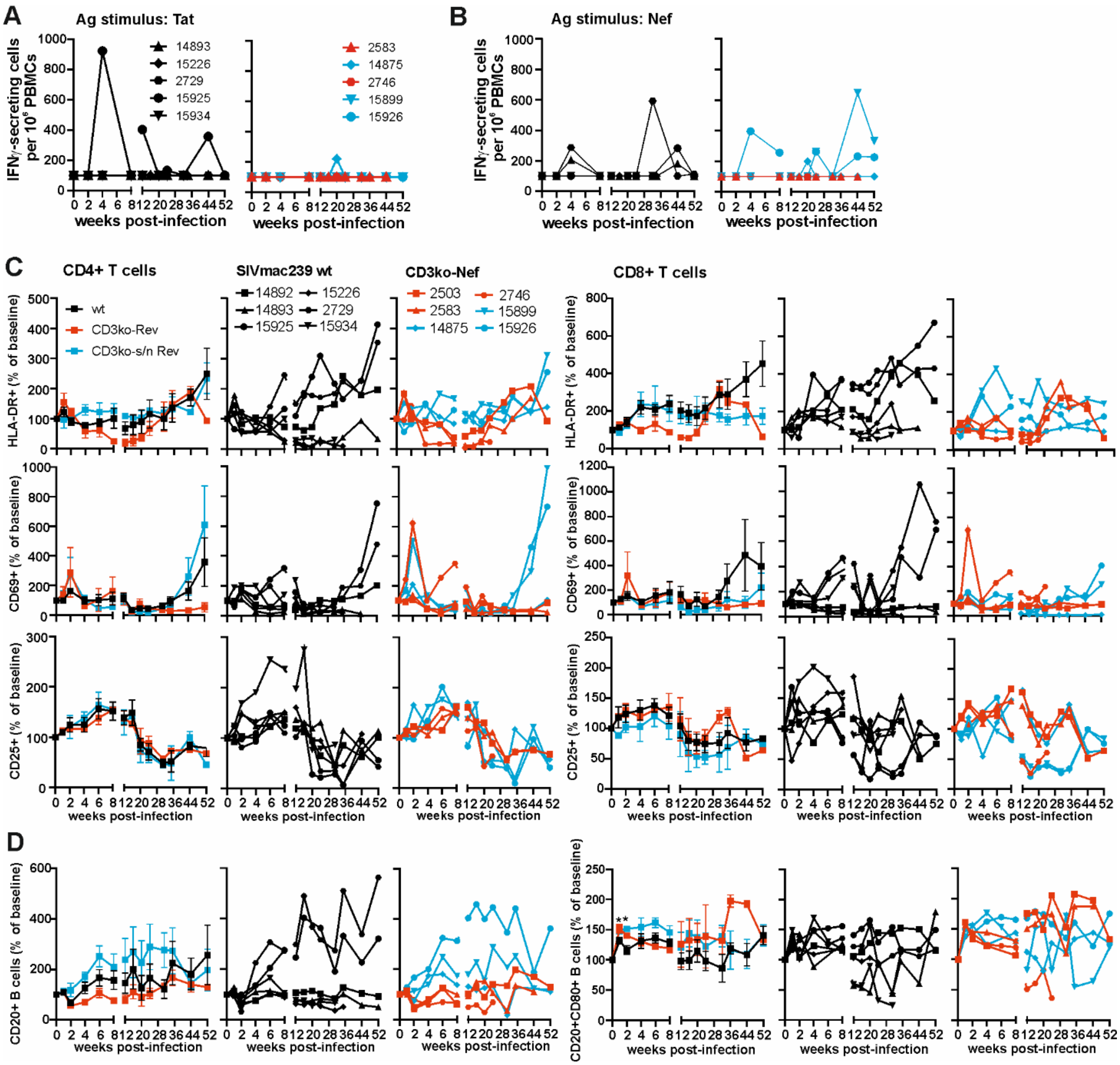

Figure S5 (related to Figure 5). Immunological findings during the first year of wt and CD3ko-Nef SIV$_{mac239}$ infection. (**A, B**) IFN-γ secreting lymphocytes as determined by ELISpot assay are shown as the number of spot-forming cells (SFC) per 10^6 PBMC after stimulation with (A) SIV-Tat or (B) SIV-Nef peptide pools. (**C**) Levels of activated HLA-DR+ (upper), CD69+ (middle) and CD25+ (bottom) CD4+ (left) and CD8+ (right) T cells in blood at the indicated weeks post-infection (wpi) relative to the baseline values (100%). The left panels show mean values (±SEM) measured for the wt (black) and CD3ko-Nef rapid (red) and no/slow (green) revertant groups and the middle and right panels values obtained for individual animals. Symbols specifying individual animals are indicated in the top left panel. (**D**) Percentages of total (left) and activated (CD80+, right) CD20+ B cells in blood at the indicated wpi relative to the baseline levels (100%).
