# Supplementary material for: Nef-Mediated CD3-TCR Downmodulation Dampens Acute Inflammation and Promotes SIV Immune Evasion
Source: Cell Rep. Author manuscript; Available in PMC 2020 Mar 3. (PMC7052273; doi:10.1016/j.celrep.2020.01.069)
Supplement: 3 [file NIHMS1563620-supplement-3.pdf]

Cell Reports

Article

Nef-Mediated CD3-TCR Downmodulation Dampens Acute Inflammation and Promotes SIV Immune Evasion

Graphical Abstract

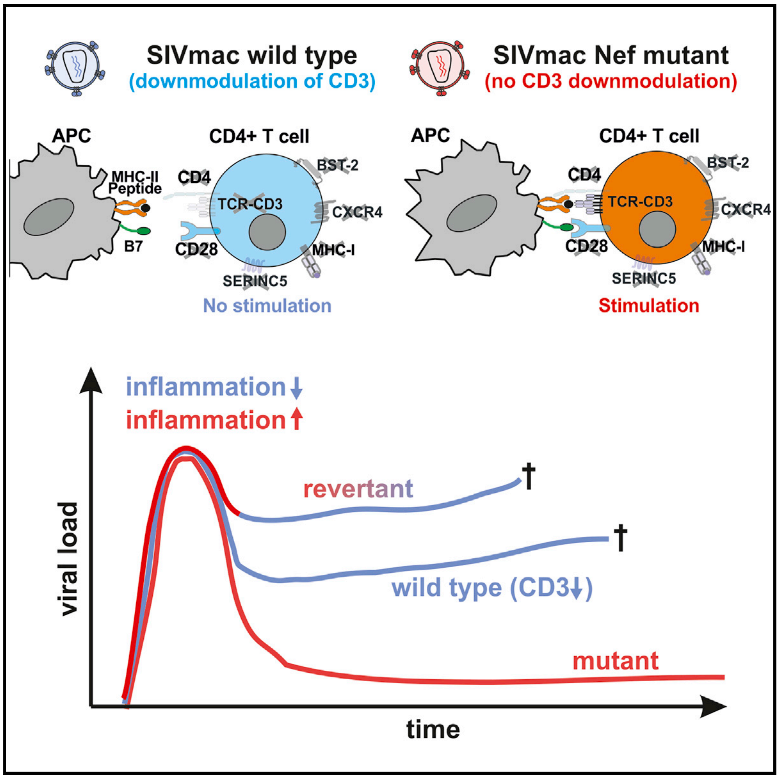

Highlights

- Mutation of I123L and L146F specifically disrupts CD3 downmodulation by SIV_{mac} Nef
- Lack of CD3 modulation by Nef increases the inflammatory response to SIV infection
- Increased immune activation aggravates the course of infection if SIV evades control
- Nef-mediated CD3 downmodulation seems critical for effective immune evasion of SIV

Authors

Simone Joas, Ulrike Sauermann, Berit Roshani, ..., Daniel Sauter, Christiane Stahl-Hennig, Frank Kirchhoff

Correspondence

frank.kirchhoff@uni-ulm.de

In Brief

HIV-1 lacks the CD3 downmodulation function of Nef that is otherwise conserved in primate lentiviruses. Joas et al. disrupted this Nef activity in SIV_{mac239} and show that Nef-mediated downmodulation of CD3 dampens inflammatory responses to SIV. This promotes effective immune evasion and maintenance of high viral loads in infected rhesus macaques.

Joas et al., 2020, Cell Reports *30*, 2261–2274
February 18, 2020 © 2020 The Author(s).
https://doi.org/10.1016/j.celrep.2020.01.069

Cell Reports
Article

OPEN ACCESS
CellPress

Nef-Mediated CD3-TCR Downmodulation Dampens Acute Inflammation and Promotes SIV Immune Evasion

Simone Joas,[1] Ulrike Sauermann,[2] Berit Roshani,[2] Antonina Klippert,[2] Maria Daskalaki,[2] Kerstin Mätz-Rensing,[2] Nicole Stolte-Leeb,[2] Anke Heigele,[1] Gregory K. Tharp,[3] Prachi Mehrotra Gupta,[3] Sydney Nelson,[3] Steven Bosinger,[3] Laura Parodi,[4] Luis Giavedoni,[4] Guido Silvestri,[3] Daniel Sauter,[1] Christiane Stahl-Hennig,[2,5] and Frank Kirchhoff[1,5,6,*]
[1]Institute of Molecular Virology – Ulm University Medical Center, Meyerhofstraße 1, 89081 Ulm, Germany
[2]German Primate Center, Kellnerweg 4, 37077 Göttingen, Germany
[3]Yerkes Primate Research Center, Emory Vaccine Center, and Department of Pathology, Emory University, Atlanta, GA, USA
[4]Host-Pathogen Interactions Program, Southwest National Primate Research Center, Texas Biomedical Research Institute, San Antonio, TX, USA
[5]These authors contributed equally
[6]Lead Contact
*Correspondence: frank.kirchhoff@uni-ulm.de
https://doi.org/10.1016/j.celrep.2020.01.069

SUMMARY

The inability of Nef to downmodulate the CD3-T cell receptor (TCR) complex distinguishes HIV-1 from other primate lentiviruses and may contribute to its high virulence. However, the role of this Nef function in virus-mediated immune activation and pathogenicity remains speculative. Here, we selectively disrupted this Nef activity in SIV_{mac239} and analyzed the consequences for the virological, immunological, and clinical outcome of infection in rhesus macaques. The inability to downmodulate CD3-TCR does not impair viral replication during acute infection but is associated with increased immune activation and antiviral gene expression. Subsequent early reversion in three of six animals suggests strong selective pressure for this Nef function and is associated with high viral loads and progression to simian AIDS. In the absence of reversions, however, viral replication and the clinical course of infection are attenuated. Thus, Nef-mediated downmodulation of CD3 dampens the inflammatory response to simian immunodeficiency virus (SIV) infection and seems critical for efficient viral immune evasion.

INTRODUCTION

T cell receptor (TCR) signaling in response to antigen recognition plays a key role in the immune response and is modulated by numerous viral pathogens (Jerome, 2008). Remarkably, primate lentiviruses show fundamental differences in their effects on TCR signaling and T cell activation. Most simian immunodeficiency viruses (SIVs), as well as HIV-2 originating from SIV_{smm}-infected sooty mangabeys, use the accessory protein Nef to remove the CD3 receptor from the cell surface (Bell et al., 1998; Iafrate et al., 1997; Schindler et al., 2006). CD3 is a key component of the TCR complex and essential for intracellular signaling as well as cell surface expression of the TCR complex. Thus, primate lentiviruses capable of downmodulating CD3 prevent the formation of the immunological synapse between virally infected $CD4^+$ T cells and antigen-presenting cells (APCs) and suppress T cell activation (Arhel et al., 2009). In stark contrast, HIV-1 and its SIV precursors infecting chimpanzees and gorillas (SIV_{cpz} and SIV_{gor}) entirely lost the CD3-TCR downmodulation function of Nef (Schindler et al., 2006). Consequently, these viruses boost rather than prevent the responsiveness of infected $CD4^+$ T cells to CD3-TCR-mediated stimulation by APCs (Arhel et al., 2009; Fenard et al., 2005; Fortin et al., 2004). The loss of this Nef function in the primate lentiviral lineage that gave rise to HIV-1 was most likely facilitated by the acquisition of a *vpu* gene (Heusinger and Kirchhoff, 2017; Kirchhoff, 2009), because Vpu and Nef-mediated downmodulation of CD3 both suppress nuclear factor κB (NF-κB)-driven antiviral gene expression (Hotter et al., 2017; Langer et al., 2019; Sauter et al., 2015).

Lack of Nef-mediated CD3 downmodulation is associated with increased levels of activation, apoptosis, and expression of death receptors and inflammatory cytokines in virally infected cultures of human $CD4^+$ T cells (Khalid et al., 2012; Schindler et al., 2006, 2008). It has been hypothesized that primate lentiviruses boosting CD3-TCR signaling and $CD4^+$ T cell activation might induce stronger immune responses than those preventing it (Kirchhoff, 2009). Indeed, chronic hyper-immune activation and high levels of apoptosis are hallmarks of pathogenic HIV-1 infection and absent in natural simian hosts of SIV that do not develop disease despite high levels of viral replication (Chahroudi et al., 2012; Sodora et al., 2009). In agreement with an increased virulence of primate lentiviruses lacking the CD3-TCR downmodulation function of Nef, SIV_{cpz} may cause an AIDS-like disease in wild chimpanzees (Keele et al., 2009). In addition, the low prevalence of other *vpu*-containing SIV strains (i.e., SIV_{gsn}, SIV_{mus}, and SIV_{mon}) in their natural host species (i.e., greater spot-nosed monkeys, mustached guenons, and mona monkeys) suggests that these viruses may also cause disease in the wild (Schmidt et al., 2017). Finally, HIV-2 is less pathogenic in humans than HIV-1 (Marlink et al., 1994), and the efficiency of Nef-mediated downmodulation of CD3 correlates with reduced

© 2020 The Author(s).
This is an open access article under the CC BY license (http://creativecommons.org/licenses/by/4.0/).

immune activation and high $CD4^+$ T cell counts in viremic HIV-2-infected individuals (Feldmann et al., 2009; Khalid et al., 2012).

While several lines of evidence support a protective role against harmful hyper-immune activation, Nef-mediated downmodulation of CD3-TCR does not prevent AIDS progression in poorly adapted recent or experimental hosts of primate lentiviruses, such as humans or rhesus macaques, respectively. A variety of mechanisms might allow well-adapted natural simian hosts to avoid chronic immune activation and remain AIDS-free despite high virus replication (Klatt et al., 2014; Paiardini et al., 2011; Palesch et al., 2018). However, even in well-adapted species such as African green monkeys and sooty mangabeys, the CD3 downmodulation function of Nef seems to reduce immune activation (Joas et al., 2018) and protect against the loss of $CD4^+$ T cells (Schindler et al., 2008). It seems conceivable that both intrinsic susceptibility of the host to disease as well as specific viral properties determine the clinical outcome of infection. Understanding the viral determinants of immune activation and inflammation is important, since these events drive disease progression and favor HIV persistence (Massanella et al., 2016). However, the potential importance of the loss of the CD3 downmodulation of Nef for the high virulence of HIV-1 has never been addressed in a disease susceptible host of primate lentiviruses. Here, we show that specific disruption of this Nef function is associated with increased acute inflammation and improved control of viral replication during the chronic phase of SIV_{mac} infection of rhesus macaques.

RESULTS

Specific Disruption of the CD3 Downmodulation Function of SIV_{mac239} Nef

Given that primate lentiviral Nef proteins differ fundamentally in their ability to downmodulate CD3-TCR cell surface expression, it seemed paradoxical that this function maps to the highly conserved core region of otherwise highly variable primate lentiviral Nef proteins (Iafrate et al., 1997; Schaefer et al., 2002). Nef performs numerous activities (Landi et al., 2011; Sauter and Kirchhoff, 2018), and for many years, it seemed impossible to disrupt the CD3 downmodulation function without affecting any other activity. Two recent advances allowed us to overcome this challenge. First, some rare, naturally occurring Nef variants in SIV_{smm}-infected sooty mangabeys are defective in CD3 downmodulation but seemed otherwise fully functional (Schindler et al., 2008; Schmökel et al., 2013). Second, recent analyses elucidated the structural basis for this Nef function and identified a hydrophobic cavity in the core domain of SIV_{mac} Nef that accommodates tyrosine-based motifs in the ζ chain of the CD3 complex for effective endocytosis (Kim et al., 2010; Manrique et al., 2017). Together, these findings suggested that specific amino acid changes located at the rim of the hydrophobic pocket in the core region of Nef specifically affect CD3-TCR downmodulation. Based on these observations, we introduced three amino acid changes (I123L, L146F and D158N) that naturally occurred in some SIV_{smm} strains, individually and in combination, into the core region of SIV_{mac239} Nef (Figure 1A). All mutant Nefs were efficiently expressed (Figure 1B), and combined changes of I123L and L146F disrupted the effect of SIV_{mac239} Nef on CD3 cell-surface expression without reducing its ability to downmodulate CD4, major histocompatibility complex (MHC) class I, CXCR4, or CD28 (Figures 1C and S1). In addition, all Nef mutants were fully active in enhancing virion infectivity (Figure 1D) and promoting viral replication in human peripheral blood mononuclear cells (PBMCs) (Figure 1E). Just like HIV-1 Nef, however, mutant SIV_{mac239} Nefs lacking the CD3 downmodulation function lost the ability to block T cell activation (Figure 1F). Altogether, our results demonstrated that combined mutations of I123L and L146F specifically disrupt the effect of SIV_{mac239} Nef on CD3, mimicking the loss of this Nef function in the SIV precursors of HIV-1.

Nef-Mediated CD3 Downmodulation Is Critical for Maintenance of High Viral Loads

Asian macaques are highly susceptible to virus-induced immunodeficiency (Evans and Silvestri, 2013), and experimental infection of rhesus macaques with SIV_{mac} is perhaps the best-characterized and most widely used non-human primate model for studies of AIDS pathogenesis, prevention, and therapy (Garcia-Tellez et al., 2016). Pioneering studies in the SIV/macaque model demonstrated that Nef is critical for efficient viral replication and full pathogenic potential (Kestler et al., 1991) and *nef*-deleted viruses were candidates for live-attenuated vaccine development (Daniel et al., 1992). This animal model also revealed strong selective pressure for intact functional *nef* genes *in vivo* (Kestler et al., 1991). In addition, infection with mutant forms of SIV_{mac239} provided insights into the relevance of specific Nef functions, such as downmodulation of the CD4 receptor or MHC class I molecules, for viral immune evasion and replication fitness (Brenner et al., 2006; Münch et al., 2001; Schindler et al., 2004; Swigut et al., 2004). For our *in vivo* studies, we used an SIV_{mac239} construct expressing a triple mutant Nef (referred to as CD3ko-Nef) containing the I123L and L146F changes that disrupt the effect on CD3 and a D158N substitution. The latter had no significant effect on Nef function (Figures 1C–1F) but was included as neutral control to better assess selective pressures on Nef and the occurrence of reversions *in vivo*.

Infection of 12 rhesus macaques with the wild-type (WT) and CD3ko-Nef SIV_{mac239} constructs (six animals each) showed that lack of CD3-TCR downmodulation of Nef has little if any effect on the kinetics and efficiency of viral replication during acute infection (Figure 2A, left). Starting from week 6, however, the virological outcome of infection diverged substantially. All six WT SIV_{mac239}-infected rhesus macaques maintained plasma viral RNA loads ranging from ~10^5 to ~10^6 copies/mL during chronic infection (Figure 2A, middle). In contrast, the levels of viremia were highly diverse in animals infected with the CD3ko-Nef SIV_{mac239} construct ranging from undetectable (i.e., $\leq$ 40 copies/mL) to almost 10^8 viral RNA copies/mL plasma (Figure 2A, right).

To define the reasons for these striking differences, we examined reversions and potential compensatory changes in *nef* from plasma viral RNA (Figures S2A and S2B). Our analyses revealed that the I123L and L146F mutations in Nef rapidly reverted between 6 and 12 weeks in animals 2503, 2583, and 2746, indicating significant selective pressure for the CD3-TCR downmodulation function (Figure 2B). These three rhesus macaques

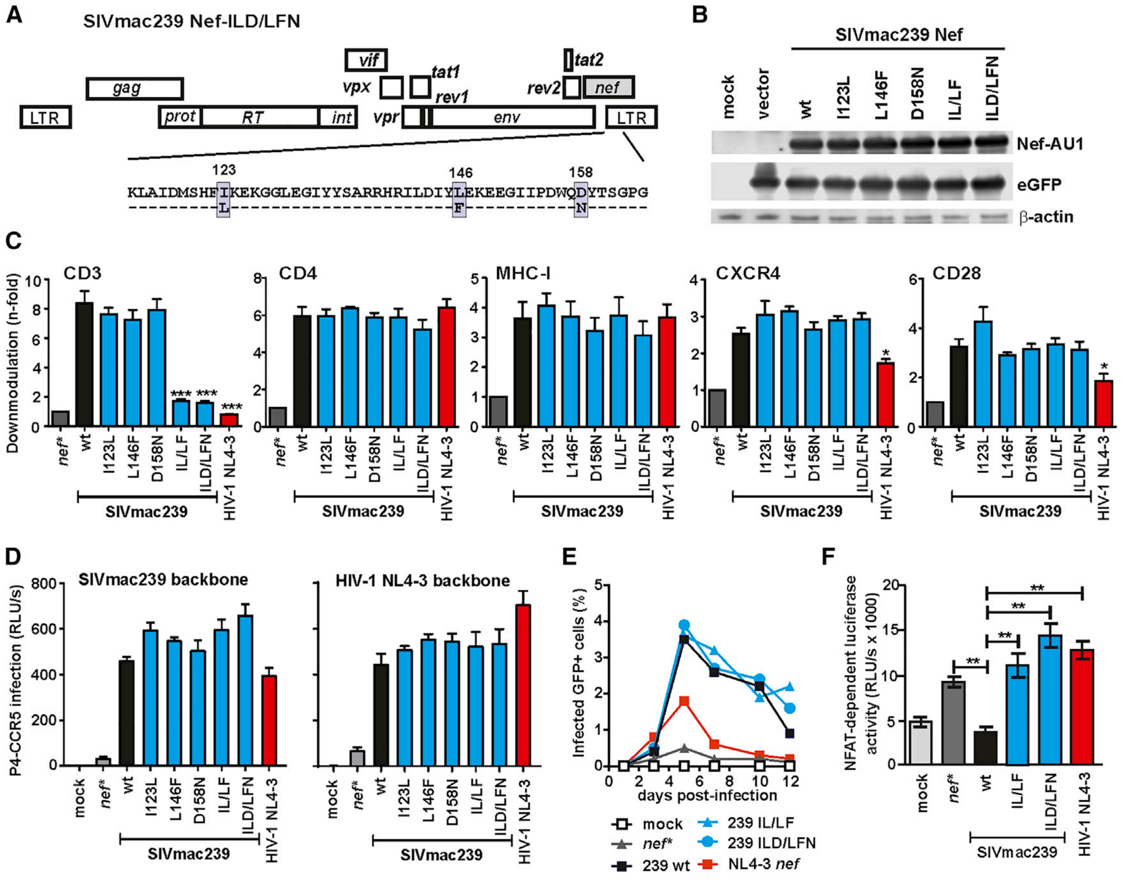

Figure 1. Specific Disruption of the CD3 Downmodulation Function of SIV_{mac239} Nef

(A) Overview on the SIV_{mac239} genome and the amino acid changes introduced into the Nef protein.

(B) Western blot analysis of HEK293T cells transfected with pCG vectors encoding AU1-tagged Nefs followed by an internal ribosome entry site (IRES) element and EGFP. The Nef variants were probed with an anti-AU1 monoclonal antibody. β-Actin and GFP were measured to control for protein quantity and transfection efficiencies, respectively.

(C) Human PBMCs were transduced with HIV-1 NL4-3 IRES-EGFP constructs expressing the indicated full-length *nef* alleles or a disrupted *nef* gene containing stop signals at codons two and three (*nef**) and assayed for surface expression of CD3, CD4, MHC-I, CXCR4, and CD28. Shown are average values + SD derived from three independent experiments. Stars indicate significant difference from WT SIV_{mac239} Nef. *p < 0.05; **p > 0.01; ***p < 0.001.

(D) Infectivity of (left) SIV_{mac239} and (right) HIV-1 NL4-3 variants expressing the indicated Nefs in P4-CCR5 reporter cells. Infections were performed in triplicate with virus stocks containing normalized amounts of p27 or p24 antigen. RLU/s, relative light units per second.

(E) Percentages of GFP^+ cells detected in PBMC cultures infected with HIV-1 NL4-3 IRES-EGFP constructs expressing the indicated Nef proteins. Shown are average values derived from three independent infections.

(F) Analysis of Jurkat cells stably transfected with an NFAT-dependent reporter gene following transduction with the indicated vesicular stomatitis virus G protein (VSV-G) pseudotyped HIV-1 Nef/EGFP constructs and subsequent stimulation with phytohemagglutinin (PHA). Levels of NFAT-dependent luciferase reporter activity are the average (± SD) of triple infections.

See also Figure S1.

showed very high levels of plasma viremia and cell-associated viral loads between 8 and 24 weeks post-infection (pi) (Figures 2C–2F). In contrast, viral replication was better controlled in animals showing slow reversion (15899 and 15926) and became undetectable in the absence of reversion (14875) (Figures 2C and 2D). Several additional amino acid changes were observed in Nef proteins from animals 15899 and 15926, including R11K, R30K, and S240T. At the experimentally mutated positions, alterations of I123V and L146C emerged in animal 15899, while in animal 15926, only the L146F and not the I123L mutation reverted (Figure S2B). Functional analyses showed that these alterations largely restored the ability of Nef to downmodulate CD3-TCR during later stages of infection (Figure S2C). Compared to WT SIV_{mac239}-infected animals, the average viral RNA loads after the acute phase (i.e., from 8 to 24 weeks pi) were 13.2-fold higher in the presence but 26.3-fold lower in the absence of reversions. In addition, lack of reversion was associated with a drop in cell-associated viral loads by approximately two orders of magnitude (Figures 2E and 2F). At later stages of infection (≥32 weeks pi), the average levels of viremia were similar in all three groups (Figure 2D). Reasons for this are the death of rapid progressors with high viral loads and the reversion of most CD3ko-Nef SIV_{mac239} constructs to the parental phenotype by that time. Altogether, our results indicate that Nef's

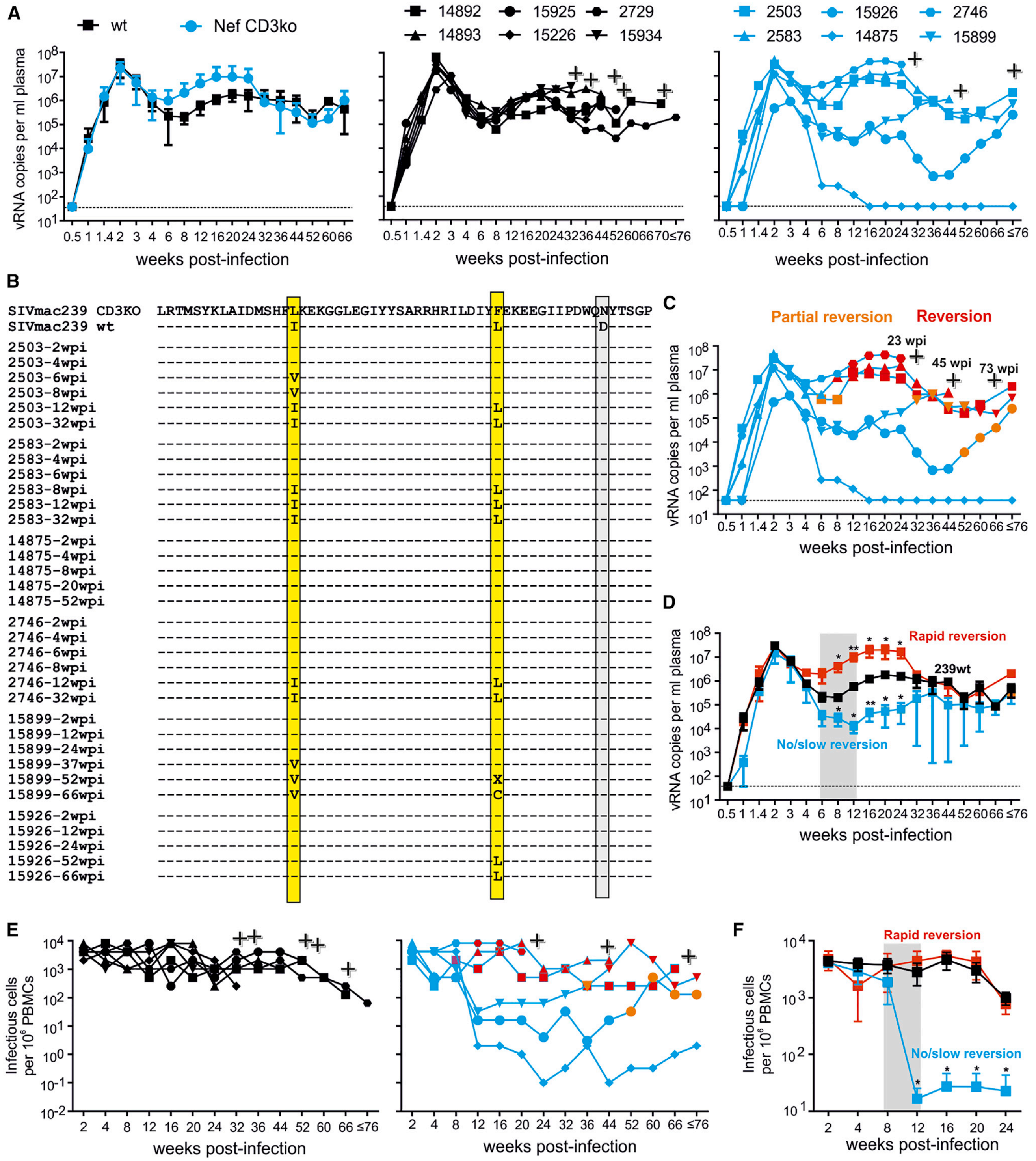

Figure 2. Importance of Nef-Mediated Downmodulation of CD3-TCR for the Maintenance of High Viral Loads in the SIV_{mac}/Macaque Model

(A) Mean viral RNA loads (± SD) in rhesus macaques infected with WT or CD3ko-Nef SIV_{mac239} constructs (n = 6 per group). The middle and right panels show viral loads in individual animals infected with the WT and CD3ko-Nef SIV_{mac239} constructs, respectively. The detection limit for viral RNA was 40 copies/mL plasma. Plus sign indicates death of the infected animal.

(B) Alterations in Nef sequences detected in plasma samples obtained from the macaques at the indicated time points. Only the predominant deduced amino acid sequence is shown; X indicates that no amino acid could be clearly assigned. The two amino acid positions involved in CD3 downmodulation are highlighted in yellow and the functionally neutral exchange in gray.

(legend continued on next page)

ability of downmodulate CD3-TCR does not affect viral replication fitness during acute infection but is critical for the maintenance of high viral loads at later stages.

Lack of CD3 Downmodulation Increases Acute Immune Activation

To determine whether lack of Nef-mediated CD3-TCR downmodulation affects the immunological response to infection, we performed RNA sequencing (RNA-seq) and cytokine array analyses of PBMCs, lymph nodes, and plasma samples, respectively. Peak levels of plasma viremia were observed at 2 weeks pi and were highly similar in WT and CD3ko-Nef SIV_{mac239} infection ($29.5 \times 10^6 \pm 8.5 \times 10^6$ versus $22.2 \times 10^6 \pm 7.1 \times 10^6$; mean viral RNA (vRNA) copies/mL ± SEM; Figure 2A). The average cell-associated viral loads in both groups were identical at this time point (Figure 2F), and reversions in *nef* were only detected several weeks later (Figure 2B). Thus, WT and CD3ko-Nef SIV_{mac239} constructs showed almost equal replication fitness during acute infection ensuring that potential differences in parameters such as immune activation were due to distinct viral properties and not differences in viral loads.

BioCarta, KEGG, and Hallmark gene set enrichment analyses (GSEAs) demonstrated that Nef-mediated downmodulation of CD3-TCR inhibits the induction of pathways involved in innate and adaptive immunity, including "inflammatory response", interleukin-1R (IL-1R), IL-17, IL-6, and Toll-like receptor (TLR) signaling as well as "antigen processing and MHC-I presentation" and "complement activation" (Figure 3A). In agreement with data obtained in cell culture (Khalid et al., 2012; Schindler et al., 2008), genes involved in NFAT and NF-κB signaling pathways were expressed at lower levels in the WT compared to the CD3ko-Nef samples. Thus, some immunosuppressive effects of Nef-mediated downmodulation of CD3-TCR may depend on reduced activity of these transcription factors.

At peak viremia (2 weeks pi), proinflammatory genes (Table S1) that are induced by TCR-CD3 stimulation in human PBMCs (Mattioli et al., 2004) were strongly enriched in animals infected with the CD3ko-Nef SIV_{mac239} construct compared to WT-infected animals (Figure 3B). This was the case for both lymph nodes and PBMCs. In particular, the expression of pentraxin-3 (PTX3), reported to regulate the inflammatory activity of macrophages (Shiraki et al., 2016), as well as IL-6 and IP-10/CXCL10, known indicators of the inflammatory status in HIV infection (de Medeiros et al., 2016), was higher in the lymph nodes of animals infected with the mutant virus than in those infected with WT SIV_{mac239} (Figure 3B). In agreement with increased immune activation, lack of Nef-mediated downmodulation of CD3-TCR was associated with strong enrichment of genes encoding restriction factors (Langer et al., 2019). Among them were many interferon (IFN)-stimulated genes (ISGs), including members of the IFIT (IFN-induced proteins with tetratricopeptide repeats) and IFITM (IFN-induced transmembrane proteins) families, ISG15, OAS1, MX1, MX2, tetherin (BST2), and IFI16 (Figure 3C). In addition, genes encoding, for example, APOBEC3 (apolipoprotein B mRNA editing enzyme catalytic polypeptide 3) family members, SLFN11, and SAMHD1 were enriched in CD3ko-Nef versus WT SIV_{mac239} infection (Figure 3C). In agreement, with the proposed role of Nef-mediated downmodulation of CD3-TCR in suppressing NF-κB activity (Heusinger and Kirchhoff, 2017), lack of this Nef function was associated with enrichment of NF-κB target genes (Table S1) in lymph nodes and PBMCs (Figure S3A). The TLR pathway was also among the top gene sets enriched in the CD3ko-Nef group compared to the WT group of animals (Figure 3A) and substantially more strongly induced in the former group during acute infection (Figure S3B).

Cytokine arrays confirmed increased induction of type I IFNs and other proinflammatory cytokines in the CD3ko-Nef compared to the WT group at 2 weeks pi, although the levels varied between individual animals (Figures 4A and 4B). Normalized for vRNA loads at 2 weeks pi, the average levels of various proinflammatory cytokines were increased between 1.8- and 6.9-fold in the CD3ko-Nef group (Figures 4B and S4A). Examination of the RNA-seq data revealed that the differences between the CD3ko-Nef and WT groups, especially in type I IFN induction, were substantially more pronounced in the lymph nodes than in blood PBMCs (Figure 4C). In line with this, several immune factors were induced to significantly higher levels by the CD3ko-Nef SIV_{mac239} construct in lymph nodes, but not in PBMCs (Figure S4B). These included IFN regulatory factor 5 (IRF5), a key regulator of inflammation involved in the induction of proinflammatory cytokines such as tumor necrosis factor alpha (TNF-α), IL-6, IL-12, and type I IFNs (Almuttaqi and Udalova, 2019); CSF2RB, a common subunit of the granulocyte macrophage colony-stimulating factor (GM-CSF), IL-3, and IL-5 receptors (Murphy and Young, 2006); and the E3 ubiquitin-protein ligase MARCH1, reported to inhibit HIV-1 (Zhang et al., 2019). Altogether, our results indicate that lack of Nef-mediated CD3 downmodulation in virally infected $CD4^+$ T cells increases the levels of acute systemic immune activation and inflammation.

Impact of Nef-Mediated CD3 Modulation on Clinical Outcome and Adaptive Immunity

Rhesus macaques are highly susceptible to SIV-induced disease, and five of the six animals infected with WT SIV_{mac239} developed simian AIDS and had to be euthanized within 80 weeks of follow-up (Table 1; Figure 5A). In contrast, the rates of clinical progression were highly diverse in macaques infected with the CD3ko-Nef SIV_{mac239} construct. In agreement with the

(C) vRNA loads in animals infected with the CD3ko-Nef SIV_{mac239} construct. Orange symbols indicate partial (i.e., one of the two critical residues), and red symbols indicate complete (i.e., both critical residues) functional reversion.

(D) Mean vRNA loads (±SEM) in macaques showing rapid or slow/now reversion compared to WT-infected animals. The shaded area indicates the time frame, in which reversions emerged in the "rapid reversion" group of animals.

(E) Cell-associated viral loads. Shown are the numbers of infectious cells per 1 million PBMC isolated from macaques infected with WT (left) or CD3ko-Nef SIV_{mac239} (right). Symbols are specified in (A) and (C), respectively.

(F) Mean cell-associated viral loads (±SEM) in macaques showing rapid (red) or slow/no (light blue) reversion compared to WT-infected animals.

See also Figure S2.

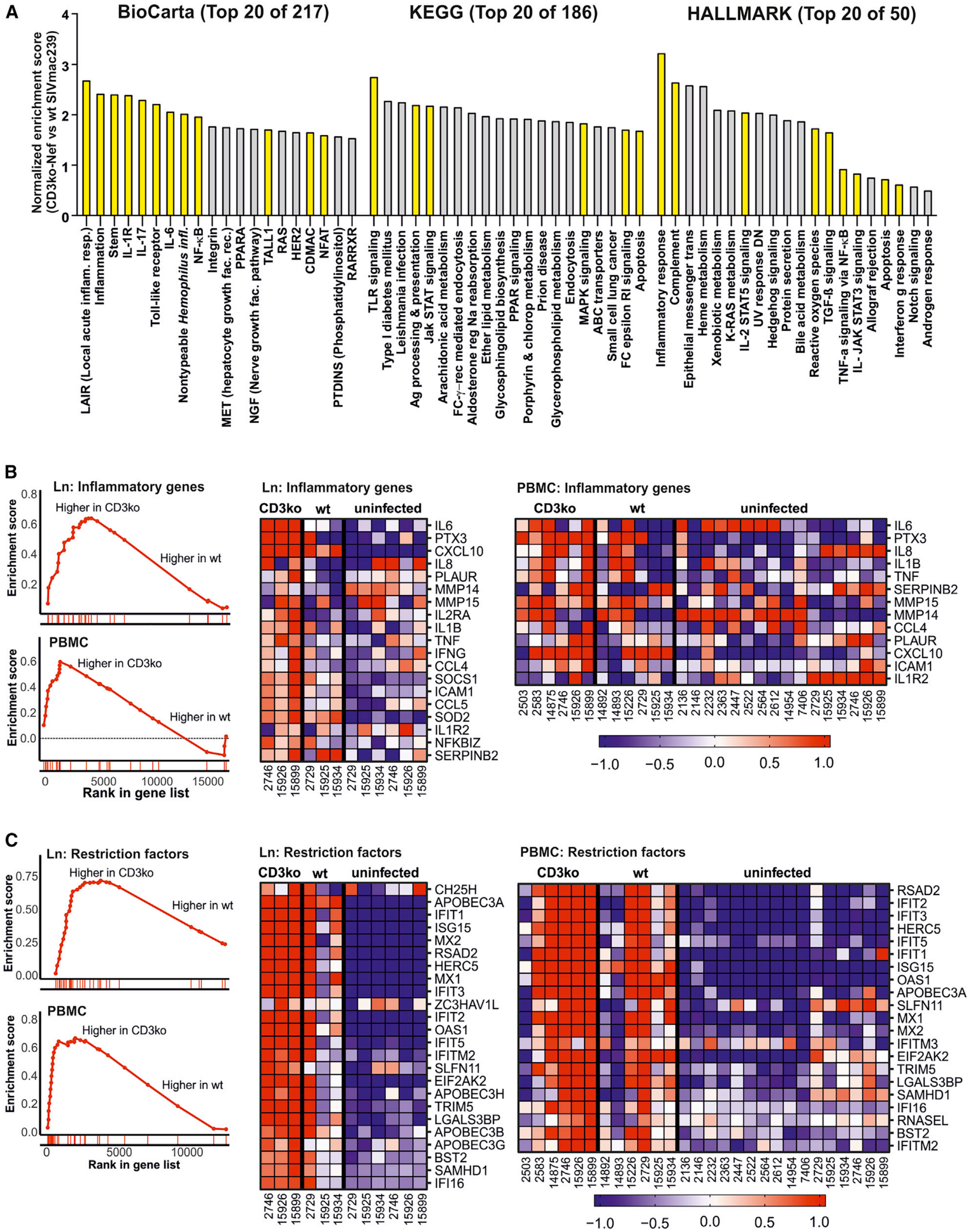

(legend on next page)

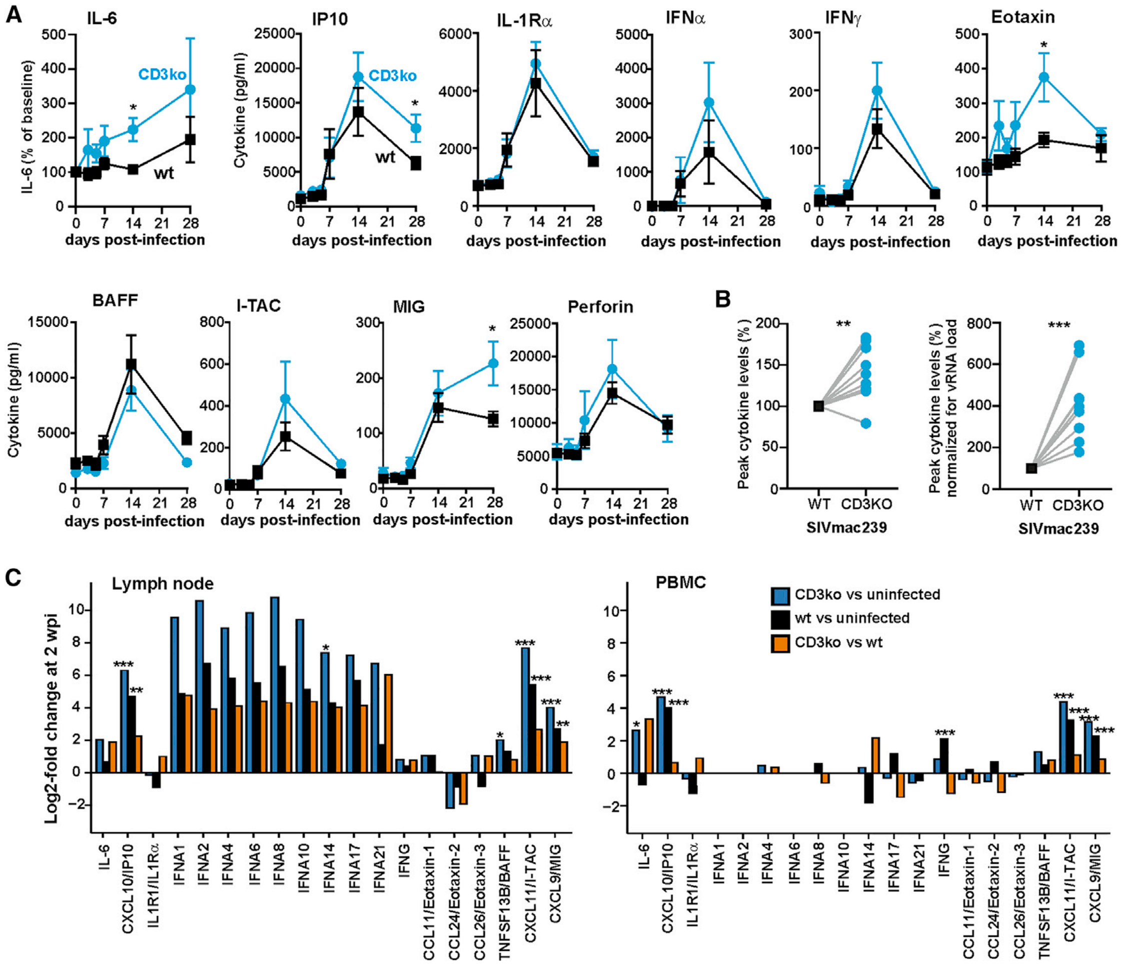

Figure 4. Expression of Proinflammatory Cytokines in WT and CD3ko-Nef SIV_{mac239} Infection

(A) Detection of proinflammatory cytokines in plasma of the 12 rhesus macaques infected with WT or CD3ko-Nef SIV_{mac239}. Cytokines were measured by Luminex at the indicated time points. The lines indicate mean ± SEM. For IL-6, induction compared to baseline is shown, since the basal levels varied substantially between animals. Asterisks in all panels indicate significant differences between the groups (*$p < 0.05$; **$p < 0.01$; ***$p < 0.001$).

(B) Comparison of the average peak levels of the cytokines indicated in (A) between the six animals infected with the CD3ko-Nef construct and macaques infected with WT SIV_{mac239} (set to 100%). In the right panel, cytokine levels were normalized for the corresponding RNA loads prior to comparison between the groups.

(C) Modulation of the mRNA levels of type I IFNs and other cytokines in lymph nodes and PBMCs isolated from animals infected with WT or CD3ko-Nef SIV_{mac239} constructs.

See also Figure S4 and Table S1.

development of high viral loads, rapid restoration of the CD3 downmodulation function of Nef in animals 2503, 2583, and 2746 was associated with progression to fatal simian AIDS by 23, 45, and 73 weeks pi (Table 1; Figure 5A). In contrast, the absence of reversions in animal 14875 was associated with undetectable viral loads and lack of disease progression (Table 1). Similarly, animal 15926 remained clinically healthy during 88 weeks of follow-up, although partial reversion ~1 year after infection coincided with increasing viral loads and mild immune activation (Figure 2A; Table 1). Finally, complete reversion

Figure 3. Lack of Nef-Mediated CD3 Downmodulation Is Associated with Increased Acute Inflammatory Responses

(A) Top 20 BioCarta, KEGG, and Hallmark pathways enriched in CD3ko-Nef compared to WT SIV_{mac239} infection of rhesus macaques. Pathways involved in intrinsic, innate, or adaptive immunity are highlighted in yellow.

(B and C) GSEA plots and heatmaps of CD3ko-Nef versus WT in lymph node and PBMC datasets for the (B) inflammatory and (C) restriction factor gene sets. The CD3ko-Nef and WT datasets consist of samples collected at 2 weeks pi from CD3ko-Nef and WT SIV_{mac239}-infected animals, respectively. The running enrichment score (y axis) is indicated for each gene ordered by their rank in the whole dataset for that specific comparison (shown by the bars below the x axis). The right panels show heatmaps for the leading-edge genes of the indicated gene sets for PBMC and lymph node (Ln) samples. The color scale is shown at the bottom, with lowest to highest gene expression across all animals represented by the blue to red color gradient

See also Figure S3 and Table S1.

Table 1. Clinical Outcome and Necropsy Findings of WT and CD3ko-Nef SIV_{mac239} Infection

Animal No.	Nef Phenotype	Reversion or Restoration[a]	Death (weeks pi)[b]	Diagnosis and Clinical Signs	Main Necropsy Findings
14892	WT	NA	70	simian AIDS; reduced feed intake 3 days before necropsy, mild diarrhea, moderate to severe anemia by week 60, severe thrombocytopenia before necropsy	erosive-ulcerative transmural jejunitis, multiple extramedullary hematopoiesis foci (indicating impaired hematopoiesis), generalized hyperplasia of lymphatic system
14893	WT	NA	51	simian AIDS; reduced feed intake for 1 week before necropsy, coughing and dyspnea by week 45, diarrhea by week 50	lymphatic system depleted, interstitial pneumonia with intranuclear inclusion bodies, suspect of CMV-induced pneumonia, subacute colitis
15226	WT	NA	35	simian AIDS; persistent diarrhea by week 25, loss of appetite by week 31	depletion of lymphatic system, severe chronic-active bacterial and parasitic gastroenteritis induced by protozoa
2729	WT	NA	89	moderate to severe thrombocytopenia by week 44, otherwise clinically healthy	chronic endocardiosis of mitral valve, generalized moderate to severe activation of lymphatic system including GALT and BALT system and kidney
15925	WT	NA	56	simian AIDS; recurrent diarrhea by week 53, reduced appetite shortly before necropsy	moderate chronic-active gastro-enteritis, generalized activation of the lymphatic system
15934	WT	NA	33	simian AIDS; mild diarrhea by week 26, moderate amount of stool protozoa, reduced feed intake and dyspnea by week 32	severe diffuse pneumocystosis of the lung
2503	CD3ko	6–12 weeks pi	73	simian AIDS; loss of body weight, loss of appetite by week 72, severe diarrhea	B cell lymphoma in the stomach, T-/B cell lymphoma in pancreoduodenal LN, tumors lymphocryptovirus positive; endoparasitosis of the large intestine by *Trichuris* and *Balantidium spec.*
2583	CD3ko	8 weeks pi	45	simian AIDS; reduced appetite by week 44, spontaneous death during the night	B cell lymphoma in the wall of the urinary bladder neck obstructing the ureters, death from uremia, tumor lymphocryptovirus positive
14875	CD3ko	none	82	clinically healthy	none
2746	CD3ko	12 weeks pi	23	simian AIDS; mild recurrent therapy-resistant diarrhea by week 19, reduced appetite	moderate chronic-active enteritis, LNs with moderate lymphoid follicular hyperplasia
15926	CD3ko	partial 52 weeks pi	88	clinically healthy	generalized mild activation of the lymphatic system
15899	CD3ko	37–52 weeks pi	81	simian AIDS; therapy-resistant watery diarrhea by week 80 with reduced appetite	moderate chronic-active gastroenteritis combined with multifocal erosive colitis induced by CMV infection, involution of the spleen with depletion of follicles

BALT, bronchus-associated lymphoid tissue; CMV, cytomegalovirus; GALT, gut-associated lymphoid tissue; LN, lymph node; NA, not applicable.
[a]Weeks pi, at which reversion was detected at amino acids positions 123 or 146 until full reversion to the WT *nef* sequence.
[b]Animals were euthanized at the end of the investigation period or when they developed severe SIV-induced immunodeficiency.

between weeks 37 and 57 in animal 15899 was associated with increasing viral loads and development of simian AIDS by the end of the study at 81 weeks pi. The total percentage of $CD4^+$ T cells in blood decreased with similar kinetics in CD3ko-Nef and WT SIV_{mac239}-infected animals and differed moderately between the rapid and slow/no reversion CD3ko-Nef groups (Figure 5B). In comparison, the numbers of $CCR5^+/CD4^+$ T cells declined rapidly in most infected animals, but this decline was more pronounced in animals showing rapid reversion of Nef function in the CD3ko-Nef group (Figure 5C).

Together, these results suggested a role of Nef-mediated CD3-TCR downmodulation in viral immune evasion during chronic infection. To obtain further insights into the underlying mechanisms, we investigated the virus-specific cellular response to three different viral peptide pools and whole chemically inactivated SIV_{mac251} particles (SIV AT-2) by IFN-γ ELISpot and the humoral response to the SIV core protein p27 and the Env protein gp130 by ELISA. All nine animals infected with the parental SIV_{mac239} construct or showing rapid reversion after infection with the CD3ko-Nef derivative showed only transient

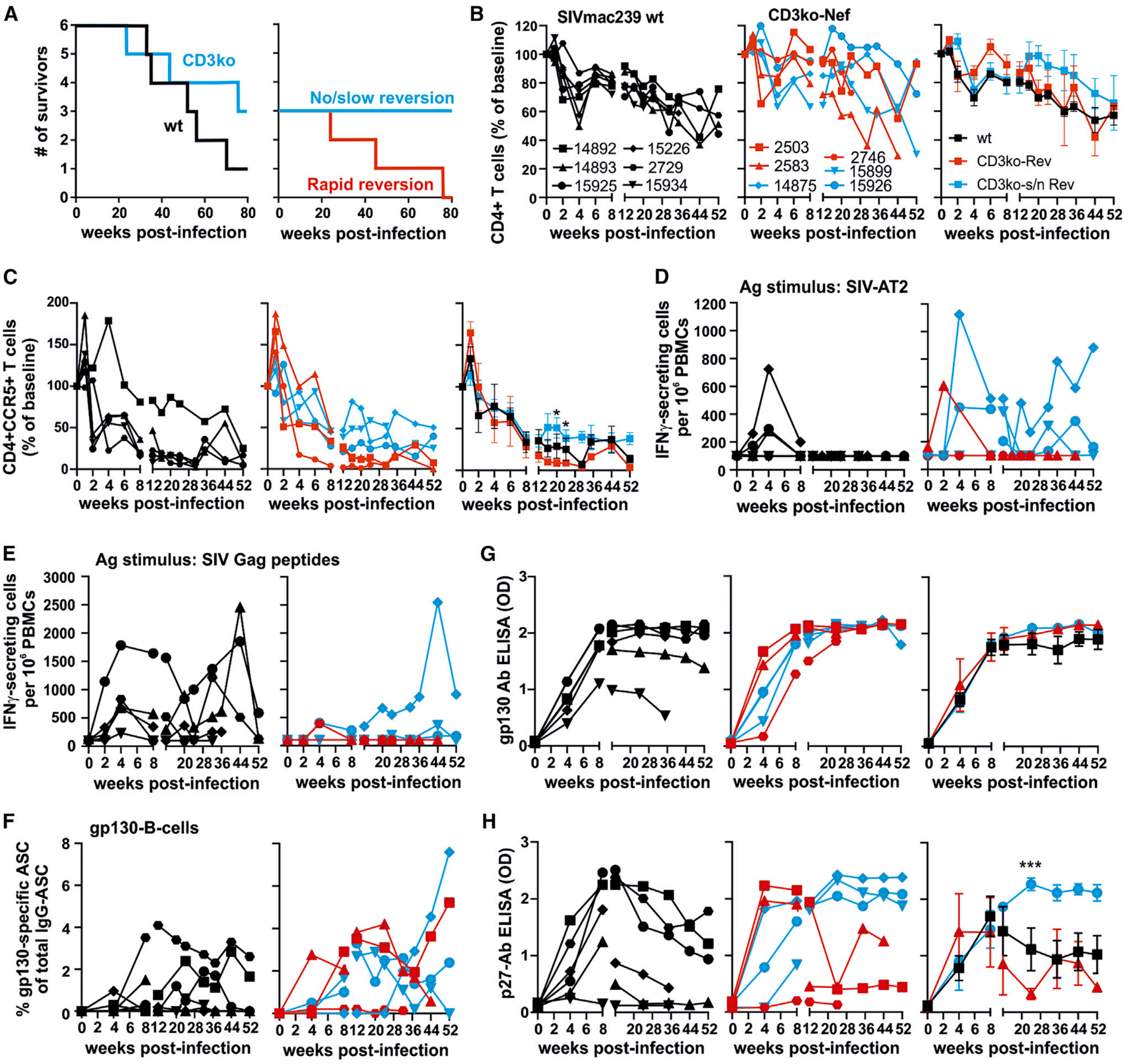

Figure 5. Survival and Immunological Outcome in WT and CD3ko-Nef SIV_{mac239} Infection

(A) Kaplan-Meier survival curves for the indicated groups of SIV-infected macaques with time measured from the date of infection.

(B and C) Levels of (B) $CD4^+$ and (C) $CCR5^+CD4^+$ T cells in blood of infected macaques compared to baseline (100%). The left and middle panels show values obtained for individual animals and the right panels mean values (± SEM) measured for the WT (black) and CD3ko-Nef rapid (red) and no/slow (light blue) revertant groups. In (C), animal 15226 was omitted, because it showed exceedingly high background levels. Asterisks in (B) and (F) indicate significant differences between the no/slow and rapid reverter groups (*$p < 0.05$; ***$p < 0.001$).

(D and E) IFN-γ ELISpot responses to (D) whole inactivated SIV_{mac} (SIV-AT2) and (E) an SIV Gag peptide pool at the indicated weeks pi. Symbols specifying individual animals are shown in (B).

(F) B cell ELISpot responses to the SIV_{mac} gp130 external envelope glycoprotein. ASC, antibody secreting cell.

(G and H) Antibody titers against the (G) SIV-gp130 Env and (H) SIV-p27 capsid proteins determined in serum of animals infected with WT or CD3ko SIV_{mac239} by ELISA (see also Figure S5).

$CD4^+$ T cell responses against SIV AT-2 during the early phase of infection (Figure 5D). In contrast, persisting responses were observed in the three rhesus macaques of the no/slow reversion group. Conversely, $CD8^+$ T-cell-mediated Gag responses were variable in macaques infected with WT SIV_{mac239} and generally weak or absent in animals that received the CD3ko-Nef mutant

virus (Figure 5E). The exception in the latter group was animal 14875 that controlled viral replication in the absence of reversions and developed a robust Gag response (Figure 5E). Significant ELISpot responses to Tat were only detected in the WT-infected animal 15925 (Figure S5A), while Nef responses were observed in the three WT SIV_{mac239}-infected animals showing relatively slow disease progression and all three no/slow reverters of the CD3ko group (Figure S5B). The levels of activated/proliferating $CD4^+$ and $CD8^+$ lymphocyte subsets varied between individual animals and did usually not differ significantly between the WT and CD3ko groups of SIV_{mac239}-infected animals (Figure S5C). Altogether, only the slow/non-progressor CD3ko group showed persistent, largely $CD4^+$ T-cell-mediated (Sauermann et al., 2017), IFN-γ responses to whole SIV antigen, while the Gag-specific $CD8^+$ T-cell-mediated responses appeared to be more prevalent in animals infected with WT SIV_{mac239}.

The percentages of B cells secreting Env-specific antibodies varied strongly within each group (Figure 5F). However, animals showing relatively slow clinical progression after WT or CD3ko-Nef SIV_{mac239} infection displayed higher levels of B cell activation than those that progressed to simian AIDS within the first year after infection (Figure S5D, left). At 1 and 2 weeks pi, B cells expressing the CD80 activation marker were more strongly induced in CD3ko-Nef than in WT SIV_{mac239} infection (Figure S5D, right). Most infected animals showed efficient and persistent humoral immune responses against the gp130 Env glycoprotein (Figure 5G). The exception was animal 15934 showing rapid disease progression after WT SIV $_{mac239}$ infection (Table 1). In comparison, the humoral response against the p27 capsid antigen was highly diverse (Figure 5H). The three animals showing rapid disease progression after WT SIV $_{mac239}$ infection displayed weak anti-p27 antibody (Ab) responses during both the acute and chronic infection. The three rapid reverters in the CD3ko group also showed weak anti-p27 Ab responses during chronic infection, although two of them (2503 and 2583) showed high reactivity during acute infection (Figure 5H). Vice versa, slow/no reversion was associated with the maintenance of high and stable anti-p27 Ab responses despite delayed and weaker reaction of two macaques (15926 and 15899) during acute infection. Thus, while the titers against gp130 Env were similar in all groups, efficient controllers had higher antibody titers against p27 than progressors, and these titers remained stable in the absence of Nef-mediated downmodulation of the TCR-CD3 receptor.

DISCUSSION

Previous studies have shown that primate lentiviral Nef proteins differ fundamentally in their effect on CD3-TCR signaling and T cell activation. Nef proteins of HIV-1 and its precursors, SIV_{cpz}, SIV_{gor}, and $SIV_{gsn/mus/mon}$, enhance the responsiveness of virally infected T cells to CD3-TCR-mediated stimulation (Fenard et al., 2005; Fortin et al., 2004; Schindler et al., 2006; Wang et al., 2000). In contrast, Nef proteins of most SIVs and HIV-2 efficiently prevent T cell activation by removing the CD3-TCR complex from the cell surface (Bell et al., 1998; Khalid et al., 2012; Schindler et al., 2006; Schmökel et al., 2011). Boosting or suppressing T cell activation may both be advantageous for primate lentiviruses depending on the immunological microenvironment. On the one hand, activated $CD4^+$ T cells are the main targets for viral replication; thus, the virus may enhance CD3-TCR signaling to render infected T cells fully permissive for viral infection and spread. On the other hand, CD3-TCR signaling is also critical for the ability of helper $CD4^+$ T cells to interact with APCs and initiate immune effector functions such as cytokine production and cytotoxicity. In the present study, we determined the impact of Nef-mediated downmodulation of CD3 on the virological, immunological, and clinical outcome of primate lentiviral infection in rhesus macaques that are highly susceptible to SIV-induced disease. We show that loss of the CD3 downmodulation function of Nef is associated with an increased acute inflammatory response to SIV infection, particularly in lymph nodes. In support of a key role in viral immune evasion, reversions in Nef that restored its ability to downmodulate CD3 coincided with the maintenance of high viral loads during chronic infection.

HIV and SIV Nef proteins perform a striking variety of functions, and dissecting their relative importance for viral fitness and pathogenicity *in vivo* requires the selectively disruption of individual activities. This task was particularly challenging for the CD3 downmodulation function, since it maps to the highly conserved core domain of Nef and active and inactive forms do not show definitive differences (Iafrate et al., 1997; Schaefer et al., 2002). While it was never possible to selectively disrupt this Nef function experimentally, the ability of lentiviruses to adapt to their host environment achieved this. Specifically, highly unusual CXCR4-tropic SIV_{smm} strains that emerged in sooty mangabeys selectively lost the CD3 downmodulation activity of Nef (Schindler et al., 2008), most likely because this activity is not advantageous in naive $CXCR4^+$ T cells (Schmökel et al., 2013). Here, we show that the I123L and L146F changes that emerged *in vivo* and disrupted the interaction of SIV_{smm} Nef with the CD3 ζ chain also specifically disrupt the CD3 downmodulation function of SIV_{mac239} Nef (Figure 1). Generation of this mutant Nef SIV_{mac239} construct allowed us to directly explore the role of Nef-mediated CD3-TCR downmodulation in primate lentivirus-mediated immune activation and pathogenesis in rhesus macaques that are highly susceptible to AIDS-like disease.

During the acute phase of infection, the CD3ko-Nef SIV_{mac239} replicated with kinetics and efficiencies that were undistinguishable from the parental virus (Figure 2A). In comparison, acute peak viral loads are usually reduced by more than two orders of magnitude upon complete deletion of *nef* in SIV_{mac239} (Janaka et al., 2018). Equal early replication fitness together with absence of reversions during acute infection allowed meaningful comparison of the immune activating properties of the CD3ko-Nef mutant and parental SIV_{mac239} strains. We found that lack of Nef-mediated CD3-TCR downmodulation further increased immune activation and the inflammatory response to infection, particularly in lymphoid tissues, confirming our hypothesis that this Nef function reduces the prevailing levels of SIV-associated immune activation. Nef-mediated downmodulation of CD3 was associated with decreased expression of restriction factors (Figure 3C) and NF-κB target genes (Figure S3A). This agrees with the evidence that Nef-mediated downmodulation of CD3-TCR

and Vpu-dependent suppression of nuclear p65 translocation represent alternative strategies to suppress NF-κB-dependent immune activation and antiviral gene expression (Heusinger and Kirchhoff, 2017). It is thought that $CD4^+$ helper T cells are most important for the adaptive immune response, i.e., efficient humoral responses and the activation of cytotoxic T lymphocytes (CTL). Thus, it is noteworthy that lack of Nef-mediated downmodulation of CD3 in infected $CD4^+$ T cells was associated with increased type I IFN induction (Figure 4) and enhanced expression of proteins involved in TLR signaling (Figure S3B), supporting a role of CD3-TCR signaling in viral immune sensing and innate immune activation. It may seem surprising that animals infected with WT and CD3ko SIV_{mac239} constructs showed similar viral loads, although the latter induced strong innate immune responses. However, infected primary $CD4^+$ T cells maintaining CD3 expression show higher levels of activation and viral gene expression (Yu et al., 2015). In addition, recent data show that lack of Nef-mediated downmodulation of CD3 is associated with increased cell-to-cell spread (Dejan Mesner and Prof. Dr. Clare Jolly, personal communication). These mechanisms might compensate for improved immune control during acute infection.

The presence or absence of reversions in Nef that restored the CD3 downmodulation function was associated with strikingly different outcomes of infection. Lack of reversions in animal 14875 was associated with efficient control of viral replication (Figure 2) and absence of disease progression (Table 1). Late reversions in macaques 15926 and 15899 were associated with increases in viral loads and slow disease progression. Surprisingly, the set-point viral loads in the three animals showing rapid reversion to a fully functional Nef were on average more than 10-fold higher compared to the six macaques infected with WT SIV_{mac239}, and all these animals progressed to simian AIDS during follow-up. One possible reason for this is that an increased acute inflammatory response provides more activated $CD4^+$ T cell targets for viral replication. Notably, it has been reported that the levels of immune activation early in HIV-1 infection are positively associated with viremia and determine the rate of $CD4^+$ T cell loss in untreated individuals (Deeks et al., 2004). In strict contrast, macaques showing no/slow restoration of the CD3 downmodulation function of Nef had ~350-fold lower set-point viral loads than rapid reverters (Figure 2D). Effective replication of the CD3ko SIV_{mac239} construct during acute infection suggests that the low levels of viremia at later stages are immune mediated rather than due to intrinsic differences in replicative capacity. In fact, no/slow reverters showed significantly stronger IFN-γ responses to whole SIV antigen (Figure 5D) and had higher antibody titers against p27 (Figure 5H) than rapid reverters. More comprehensive studies are required to fully define the mechanisms underlying effective immune control in the absence of Nef-mediated downmodulation of CD3, but our preliminary data suggest that lack of this Nef function is associated with stronger $CD4^+$ T cells responses.

A role of CD3 downmodulation in both viral pathogenesis and immune evasion agrees with early findings that chimeric SIV_{mac239} constructs expressing HIV-1 *nef* genes frequently result in an ‘‘all or nothing’’ phenotype, i.e., high viral loads and rapid disease progression or effective control of the chimeric viruses (Alexander et al., 1999; Kirchhoff et al., 1999; Mandell et al., 1999). In agreement with the present data, SIV_{agm} constructs expressing an HIV-1 *nef* gene showed high levels of replication during acute infection but were subsequently better controlled by the immune system than the parental SIV_{agm} construct expressing a Nef that downmodulates CD3-TCR (Joas et al., 2018). In these experiments, insertion of a functional *vpu* gene into the SIV_{agm} genome in addition to the HIV-1 *nef* gene was associated with ~20-fold higher viral loads during chronic infection. Thus, Vpu may functionally compensate for the lack of the CD3 downmodulation function of Nef. Altogether, our results further support that enhanced immune activation and type I IFN induction may have both beneficial and detrimental effects (Utay and Douek, 2016), the former if leading to effective control of virus replication and the latter if the virus is capable of efficiently evading immune control and using the increased availability of activated $CD4^+$ target T cells to its advantage.

In summary, we show for the first time that specific disruption of the CD3 downmodulation function of Nef that was lost by HIV-1 and its SIV precursors but is otherwise highly conserved among primate lentiviruses affects the systemic acute immune response and the virological and clinical outcomes of SIV_{mac} infection in rhesus macaques. Specifically, our data demonstrate that lack of CD3-TCR downmodulation in SIV-infected cells clearly increases inflammation and immune activation *in vivo*. Our results further suggest that the CD3-TCR downmodulation function of Nef is essential for efficient viral immune evasion and maintenance of high viral loads in primate lentiviruses lacking a *vpu* gene.

STAR★METHODS

Detailed methods are provided in the online version of this paper and include the following:

- KEY RESOURCES TABLE
- LEAD CONTACT AND MATERIALS AVAILABILITY
- EXPERIMENTAL MODEL AND SUBJECT DETAILS
  - Ethical statement for human samples
  - Ethical statement for animal experiments
  - Cell lines
  - Primary cell culture
- METHOD DETAILS
  - Proviral constructs
  - Expression vectors
  - Western blot
  - Flow cytometry
  - Virus stocks and transductions
  - Viral infectivity
  - NF-AT assay
  - Experimental animals, virus inoculation and specimen collection
  - RNA Purification
  - Viral RNA and cell-associated viral load quantitation
  - ELISpot assay
  - B cell ELISpot
  - Serological testing
  - MHC typing

- ○ Transcriptome analysis
- ○ Gene set enrichment analysis and heatmaps
- ○ Multiplex cytokine profile analysis
- QUANTIFICATION AND STATISTICAL ANALYSIS
- DATA AND CODE AVAILABILITY

SUPPLEMENTAL INFORMATION

Supplemental Information can be found online at https://doi.org/10.1016/j.celrep.2020.01.069.

ACKNOWLEDGMENTS

We thank Kerstin Regensburger, Daniela Krnavek, Martha Mayer, Regina Burger, Susanne Engelhart, Nicole Leuchte, and Sandra Heine for excellent technical assistance; Jan Schmökel for support during the early stage of the project; and Matthias Mietsch for skilled and comprehensive veterinary support. The following reagents were obtained from the Centre for AIDS Reagents, NIBSC, UK: SIV_{mac239} gp130 His recombinant protein (ARP100841) from Dr. Klaus Überla (via the NIH AIDS Reagent Program, Division of AIDS, NIAID, NIH); recombinant SIV_{mac251} p27 (ARP643), Aldrithiol-2-treated SIV_{mac239}*/SuPT-1CCFR5 CL.30/Aldrithiol-2-treated SuPT1-CCR5 CL.30 microvesicles (ARP1018) (donated by the AIDS and Cancer Virus Program/Biological Products Core, National Cancer Institute, USA); and SIV Gag (EVA7066), Nef (EVA777), and Tat (EVA 7069) overlapping peptides. This work was supported by grants from the German Research Foundation (DFG CRC 1279 and SPP 1923) and used resources supported by the Southwest National Primate Research Center grant P51 OD011133 from the Office of Research Infrastructure Programs, National Institutes of Health. The funders had no role in study design, data collection and analysis, decision to publish, or preparation of the manuscript.

AUTHOR CONTRIBUTIONS

S.J. performed most of the *in vitro* experiments. A.H. also provided experimental data and generated mutant Nef constructs. U.S., B.R., A.K., M.D., K.M.-R., N.S.-L., and C.S.-H. performed the *in vivo* analyses in the SIV/macaque model. G.K.T., S.N., P.M.G., and S.B. performed the transcriptome analyses, and L.P. and L.G. determined the multiplex cytokine profiles. S.B., L.G., G.S., D.S., C.S.-H., and F.K. conceived and designed the experiments and analyzed the data. F.K. planned the study and wrote the initial draft manuscript. All authors have seen, edited, and approved the final version of the manuscript.

DECLARATION OF INTERESTS

The authors declare no competing interests.

Received: August 27, 2019
Revised: December 10, 2019
Accepted: January 21, 2020
Published: February 18, 2020

REFERENCES

Alexander, L., Du, Z., Howe, A.Y., Czajak, S., and Desrosiers, R.C. (1999). Induction of AIDS in rhesus monkeys by a recombinant simian immunodeficiency virus expressing nef of human immunodeficiency virus type 1. J. Virol. *73*, 5814–5825.

Almuttaqi, H., and Udalova, I.A. (2019). Advances and challenges in targeting IRF5, a key regulator of inflammation. FEBS J. *286*, 1624–1637.

Anders, S., Pyl, P.T., and Huber, W. (2015). HTSeq—a Python framework to work with high-throughput sequencing data. Bioinformatics *31*, 166–169.

Arhel, N., Lehmann, M., Clauss, K., Nienhaus, G.U., Piguet, V., and Kirchhoff, F. (2009). The inability to disrupt the immunological synapse between infected human T cells and APCs distinguishes HIV-1 from most other primate lentiviruses. J. Clin. Invest. *119*, 2965–2975.

Bell, I., Ashman, C., Maughan, J., Hooker, E., Cook, F., and Reinhart, T.A. (1998). Association of simian immunodeficiency virus Nef with the T-cell receptor (TCR) zeta chain leads to TCR down-modulation. J. Gen. Virol. *79*, 2717–2727.

Brenner, M., Münch, J., Schindler, M., Wildum, S., Stolte, N., Stahl-Hennig, C., Fuchs, D., Mätz-Rensing, K., Franz, M., Heeney, J., et al. (2006). Importance of the N-distal AP-2 binding element in Nef for simian immunodeficiency virus replication and pathogenicity in rhesus macaques. J. Virol. *80*, 4469–4481.

Chahroudi, A., Bosinger, S.E., Vanderford, T.H., Paiardini, M., and Silvestri, G. (2012). Natural SIV hosts: showing AIDS the door. Science *335*, 1188–1193.

Charneau, P., Mirambeau, G., Roux, P., Paulous, S., Buc, H., and Clavel, F. (1994). HIV-1 reverse transcription. A termination step at the center of the genome. J. Mol. Biol. *241*, 651–662.

Daniel, M., Kirchhoff, F., Czajak, S., Sehgal, P., and Desrosiers, R. (1992). Protective effects of a live attenuated SIV vaccine with a deletion in the nef gene. Science *258*, 1938–1941.

de Medeiros, R.M., Valverde-Villegas, J.M., Junqueira, D.M., Gräf, T., Lindenau, J.D., de Mello, M.G., Vianna, P., Almeida, S.E., and Chies, J.A. (2016). Rapid and Slow Progressors Show Increased IL-6 and IL-10 Levels in the Pre-AIDS Stage of HIV Infection. PLoS One *11*, e0156163. https://doi.org/10.1371/journal.pone.0156163.

Deeks, S.G., Kitchen, C.M.R., Liu, L., Guo, H., Gascon, R., Narváez, A.B., Hunt, P., Martin, J.N., Kahn, J.O., Levy, J., et al. (2004). Immune activation set point during early HIV infection predicts subsequent CD4+ T-cell changes independent of viral load. Blood *104*, 942–947.

Dobin, A., Davis, C.A., Schlesinger, F., Drenkow, J., Zaleski, C., Jha, S., Batut, P., Chaisson, M., and Gingeras, T.R. (2013). STAR: ultrafast universal RNA-seq aligner. Bioinformatics *29*, 15–21.

DuBridge, R.B., Tang, P., Hsia, H.C., Leong, P.M., Miller, J.H., and Calos, M.P. (1987). Analysis of mutation in human cells by using an Epstein-Barr virus shuttle system. Mol. Cell. Biol. *7*, 379–387.

Evans, D.T., and Silvestri, G. (2013). Nonhuman primate models in AIDS research. Curr. Opin. HIV AIDS *8*, 255–261.

Feldmann, J., Leligdowicz, A., Jaye, A., Dong, T., Whittle, H., and Rowland-Jones, S.L. (2009). Downregulation of the T-cell receptor by human immunodeficiency virus type 2 Nef does not protect against disease progression. J. Virol. *83*, 12968–12972.

Fenard, D., Yonemoto, W., de Noronha, C., Cavrois, M., Williams, S.A., and Greene, W.C. (2005). Nef is physically recruited into the immunological synapse and potentiates T cell activation early after TCR engagement. J. Immunol. *175*, 6050–6057.

Fortin, J.F., Barat, C., Beauséjour, Y., Barbeau, B., and Tremblay, M.J. (2004). Hyper-responsiveness to stimulation of human immunodeficiency virus-infected CD4+ T cells requires Nef and Tat virus gene products and results from higher NFAT, NF-kappaB, and AP-1 induction. J. Biol. Chem. *279*, 39520–39531.

Garcia-Tellez, T., Huot, N., Ploquin, M.J., Rascle, P., Jacquelin, B., and Müller-Trutwin, M. (2016). Non-human primates in HIV research: achievements, limits and alternatives. Infect. Genet. Evol. *46*, 324–332.

Giavedoni, L.D. (2005). Simultaneous detection of multiple cytokines and chemokines from nonhuman primates using luminex technology. J. Immunol. Methods *301*, 89–101.

Gundlach, B.R., Reiprich, S., Sopper, S., Means, R.E., Dittmer, U., Mätz-Rensing, K., Stahl-Hennig, C., and Uberla, K. (1998). Env-independent protection induced by live, attenuated simian immunodeficiency virus vaccines. J. Virol. *72*, 7846–7851.

Heusinger, E., and Kirchhoff, F. (2017). Primate lentiviruses modulate NF-κB activity by multiple mechanisms to fine-tune viral and cellular gene expression. Front. Microbiol. *8*, 198.

Hotter, D., Krabbe, T., Reith, E., Gawanbacht, A., Rahm, N., Ayouba, A., Van Driessche, B., Van Lint, C., Peeters, M., Kirchhoff, F., and Sauter, D. (2017).

Primate lentiviruses use at least three alternative strategies to suppress NF-κB-mediated immune activation. PLoS Pathog. *13*, e1006598.

Iafrate, A.J., Bronson, S., and Skowronski, J. (1997). Separable functions of Nef disrupt two aspects of T cell receptor machinery: CD4 expression and CD3 signaling. EMBO J. *16*, 673–684.

Janaka, S.K., Tavakoli-Tameh, A., Neidermyer, W.J., Jr., Serra-Moreno, R., Hoxie, J.A., Desrosiers, R.C., Johnson, R.P., Lifson, J.D., Wolinsky, S.M., and Evans, D.T. (2018). Polymorphisms in rhesus macaque tetherin are associated with differences in acute viremia in simian immunodeficiency virus Δ*nef*-infected animals. J. Virol. *92*, e00542-18.

Jerome, K.R. (2008). Viral modulation of T-cell receptor signaling. J. Virol. *82*, 4194–4204.

Joas, S., Parrish, E.H., Gnanadurai, C.W., Lump, E., Stürzel, C.M., Parrish, N.F., Learn, G.H., Sauermann, U., Neumann, B., Rensing, K.M., et al. (2018). Species-specific host factors rather than virus-intrinsic virulence determine primate lentiviral pathogenicity. Nat. Commun. *9*, 1371.

Keele, B.F., Jones, J.H., Terio, K.A., Estes, J.D., Rudicell, R.S., Wilson, M.L., Li, Y., Learn, G.H., Beasley, T.M., Schumacher-Stankey, J., et al. (2009). Increased mortality and AIDS-like immunopathology in wild chimpanzees infected with SIVcpz. Nature *460*, 515–519.

Kestler, H.W., 3rd, Ringler, D.J., Mori, K., Panicali, D.L., Sehgal, P.K., Daniel, M.D., and Desrosiers, R.C. (1991). Importance of the nef gene for maintenance of high virus loads and for development of AIDS. Cell *65*, 651–662.

Khalid, M., Yu, H., Sauter, D., Usmani, S.M., Schmokel, J., Feldman, J., Gruters, R.A., van der Ende, M.E., Geyer, M., Rowland-Jones, S., et al. (2012). Efficient Nef-mediated downmodulation of TCR-CD3 and CD28 is associated with high CD4+ T cell counts in viremic HIV-2 infection. J. Virol. *86*, 4906–4920.

Kim, W.M., Sigalov, A.B., and Stern, L.J. (2010). Pseudo-merohedral twinning and noncrystallographic symmetry in orthorhombic crystals of SIVmac239 Nef core domain bound to different-length TCRζ fragments. Acta Crystallogr. D. Biol. Crystallogr. *66*, 163–175.

Kirchhoff, F. (2009). Is the high virulence of HIV-1 an unfortunate coincidence of primate lentiviral evolution? Nat. Rev. Microbiol. *7*, 467–476.

Kirchhoff, F., Münch, J., Carl, S., Stolte, N., Mätz-Rensing, K., Fuchs, D., Haaft, P.T., Heeney, J.L., Swigut, T., Skowronski, J., and Stahl-Hennig, C. (1999). The human immunodeficiency virus type 1 nef gene can to a large extent replace simian immunodeficiency virus nef in vivo. J. Virol. *73*, 8371–8383.

Klatt, N.R., Bosinger, S.E., Peck, M., Richert-Spuhler, L.E., Heigele, A., Gile, J.P., Patel, N., Taaffe, J., Julg, B., Camerini, D., et al. (2014). Limited HIV infection of central memory and stem cell memory CD4+ T cells is associated with lack of progression in viremic individuals. PLoS Pathog. *10*, e1004345. https://doi.org/10.1371/journal.ppat.1004345.

Krapp, C., Hotter, D., Gawanbacht, A., McLaren, P.J., Kluge, S.F., Stürzel, C.M., Mack, K., Reith, E., Engelhart, S., Ciuffi, A., et al. (2016). Guanylate binding protein (GBP) 5 is an interferon-inducible inhibitor of HIV-1 infectivity. Cell Host Microbe *19*, 504–514.

Landi, A., Iannucci, V., Nuffel, A.V., Meuwissen, P., and Verhasselt, B. (2011). One protein to rule them all: modulation of cell surface receptors and molecules by HIV Nef. Curr. HIV. Res. *9*, 496–504.

Langer, S., Hammer, C., Hopfensperger, K., Klein, L., Hotter, D., De Jesus, P.D., Herbert, K.M., Pache, L., Smith, N., van der Merwe, J.A., et al. (2019). HIV-1 Vpu is a potent transcriptional suppressor of NF-κB-elicited antiviral immune responses. eLife *8*, e41930.

Love, M.I., Huber, W., and Anders, S. (2014). Moderated estimation of fold change and dispersion for RNA-seq data with DESeq2. Genome Biol. *15*, 550.

Mandell, C.P., Reyes, R.A., Cho, K., Sawai, E.T., Fang, A.L., Schmidt, K.A., and Luciw, P.A. (1999). SIV/HIV Nef recombinant virus (SHIVnef) produces simian AIDS in rhesus macaques. Virology *265*, 235–251.

Manrique, S., Sauter, D., Horenkamp, F.A., Lülf, S., Yu, H., Hotter, D., Anand, K., Kirchhoff, F., and Geyer, M. (2017). Endocytic sorting motif interactions involved in Nef-mediated downmodulation of CD4 and CD3. Nat. Commun. *8*, 442.

Marlink, R., Kanki, P., Thior, I., Travers, K., Eisen, G., Siby, T., Traore, I., Hsieh, C., Dia, M., Gueye, E., et al. (1994). Reduced rate of disease development after HIV-2 infection as compared to HIV-1. Science *265*, 1587–1590.

Massanella, M., Fromentin, R., and Chomont, N. (2016). Residual inflammation and viral reservoirs: alliance against an HIV cure. Curr. Opin. HIV AIDS *11*, 234–241.

Mattioli, I., Dittrich-Breiholz, O., Livingstone, M., Kracht, M., and Schmitz, M.L. (2004). Comparative analysis of T-cell costimulation and CD43 activation reveals novel signaling pathways and target genes. Blood *104*, 3302–3304.

Münch, J., Stolte, N., Fuchs, D., Stahl-Hennig, C., and Kirchhoff, F. (2001). Efficient class I major histocompatibility complex down-regulation by simian immunodeficiency virus Nef is associated with a strong selective advantage in infected rhesus macaques. J. Virol. *75*, 10532–10536.

Münch, J., Rajan, D., Schindler, M., Specht, A., Rücker, E., Novembre, F.J., Nerrienet, E., Müller-Trutwin, M.C., Peeters, M., Hahn, B.H., and Kirchhoff, F. (2007). Nef-mediated enhancement of virion infectivity and stimulation of viral replication are fundamental properties of primate lentiviruses. J. Virol. *81*, 13852–13864.

Murphy, J.M., and Young, I.G. (2006).). IL-3, IL-5, and GM-CSF signaling: crystal structure of the human beta-common receptor. Vitam. Horm. *74*, 1–30.

Norley, S.G., Löwer, J., and Kurth, R. (1993). Insufficient inactivation of HIV-1 in human cryo poor plasma by beta-propiolactone: results from a highly accurate virus detection method. Biologicals *21*, 251–258.

Paiardini, M., Cervasi, B., Reyes-Aviles, E., Micci, L., Ortiz, A.M., Chahroudi, A., Vinton, C., Gordon, S.N., Bosinger, S.E., Francella, N., et al. (2011). Low levels of SIV infection in sooty mangabey central memory CD^{4+} T cells are associated with limited CCR5 expression. Nat. Med. *17*, 830–836.

Palesch, D., Bosinger, S.E., Tharp, G.K., Vanderford, T.H., Paiardini, M., Chahroudi, A., Johnson, Z.P., Kirchhoff, F., Hahn, B.H., Norgren, R.B., et al. (2018). Sooty mangabey genome sequence provides insight into AIDS resistance in a natural SIV host. Nature *553*, 77–81.

Pantoja, P., Pérez-Guzmán, E.X., Rodríguez, I.V., White, L.J., González, O., Serrano, C., Giavedoni, L., Hodara, V., Cruz, L., Arana, T., et al. (2017). Zika virus pathogenesis in rhesus macaques is unaffected by pre-existing immunity to dengue virus. Nat. Commun. *8*, 15674.

Sauermann, U., Siddiqui, R., Suh, Y.S., Platzer, M., Leuchte, N., Meyer, H., Mätz-Rensing, K., Stoiber, H., Nürnberg, P., Hunsmann, G., et al. (2008). Mhc class I haplotypes associated with survival time in simian immunodeficiency virus (SIV)-infected rhesus macaques. Genes Immun. *9*, 69–80.

Sauermann, U., Radaelli, A., Stolte-Leeb, N., Raue, K., Bissa, M., Zanotto, C., Krawczak, M., Tenbusch, M., Überla, K., Keele, B.F., et al. (2017). Vector order determines protection against pathogenic simian immunodeficiency virus infection in a triple-component vaccine by balancing $CD4^{+}$ and $CD8^{+}$ T-cell responses. J. Virol. *91*, e01120-17.

Sauter, D., and Kirchhoff, F. (2018). Multilayered and versatile inhibition of cellular antiviral factors by HIV and SIV accessory proteins. Cytokine Growth Factor Rev. *40*, 3–12.

Sauter, D., Schindler, M., Specht, A., Landford, W.N., Münch, J., Kim, K.A., Votteler, J., Schubert, U., Bibollet-Ruche, F., Keele, B.F., et al. (2009). Tetherin-driven adaptation of Vpu and Nef function and the evolution of pandemic and nonpandemic HIV-1 strains. Cell Host Microbe *6*, 409–421.

Sauter, D., Hotter, D., Van Driessche, B., Stürzel, C.M., Kluge, S.F., Wildum, S., Yu, H., Baumann, B., Wirth, T., Plantier, J.C., et al. (2015). Differential regulation of NF-κB-mediated proviral and antiviral host gene expression by primate lentiviral Nef and Vpu proteins. Cell Rep. *10*, 586–599.

Schaefer, T.M., Bell, I., Pfeifer, M.E., Ghosh, M., Trible, R.P., Fuller, C.L., Ashman, C., and Reinhart, T.A. (2002). The conserved process of TCR/CD3 complex down-modulation by SIV Nef is mediated by the central core, not endocytic motifs. Virology *302*, 106–122.

Schindler, M., Würfl, S., Benaroch, P., Greenough, T.C., Daniels, R., Easterbrook, P., Brenner, M., Münch, J., and Kirchhoff, F. (2003). Down-modulation of mature major histocompatibility complex class II and up-regulation of

invariant chain cell surface expression are well-conserved functions of human and simian immunodeficiency virus nef alleles. J. Virol. *77*, 10548–10556.

Schindler, M., Münch, J., Brenner, M., Stahl-Hennig, C., Skowronski, J., and Kirchhoff, F. (2004). Comprehensive analysis of nef functions selected in simian immunodeficiency virus-infected macaques. J. Virol. *78*, 10588–10597.

Schindler, M., Münch, J., Kutsch, O., Li, H., Santiago, M.L., Bibollet-Ruche, F., Müller-Trutwin, M.C., Novembre, F.J., Peeters, M., Courgnaud, V., et al. (2006). Nef-mediated suppression of T cell activation was lost in a lentiviral lineage that gave rise to HIV-1. Cell *125*, 1055–1067.

Schindler, M., Schmökel, J., Specht, A., Li, H., Münch, J., Khalid, M., Sodora, D.L., Hahn, B.H., Silvestri, G., and Kirchhoff, F. (2008). Inefficient Nef-mediated downmodulation of CD3 and MHC-I correlates with loss of CD4+T cells in natural SIV infection. PLoS Pathog. *4*, e1000107.

Schmidt, F., Liegeois, F., Greenwood, E.J.D., LeBreton, M., Lester, J., Deleplancque, L., Peeters, M., Aghokeng, A., Tamoufe, U., Diffo, J.L.D., et al. (2017). Phyloepidemiological analysis reveals that viral divergence led to the paucity of simian immunodeficiency virus SIVmus/gsn/mon infections in wild populations. J. Virol. *91*, e01884-16.

Schmökel, J., Sauter, D., Schindler, M., Leendertz, F.H., Bailes, E., Dazza, M.C., Saragosti, S., Bibollet-Ruche, F., Peeters, M., Hahn, B.H., and Kirchhoff, F. (2011). The presence of a vpu gene and the lack of Nef-mediated downmodulation of T cell receptor-CD3 are not always linked in primate lentiviruses. J. Virol. *85*, 742–752.

Schmökel, J., Li, H., Shabir, A., Yu, H., Geyer, M., Silvestri, G., Sodora, D.L., Hahn, B.H., and Kirchhoff, F. (2013). Link between primate lentiviral coreceptor usage and Nef function. Cell Rep. *5*, 997–1009.

Shiraki, A., Kotooka, N., Komoda, H., Hirase, T., Oyama, J.I., and Node, K. (2016). Pentraxin-3 regulates the inflammatory activity of macrophages. Biochem. Biophys. Rep. *5*, 290–295.

Sodora, D.L., Allan, J.S., Apetrei, C., Brenchley, J.M., Douek, D.C., Else, J.G., Estes, J.D., Hahn, B.H., Hirsch, V.M., Kaur, A., et al. (2009). Toward an AIDS vaccine: lessons from natural simian immunodeficiency virus infections of African nonhuman primate hosts. Nat. Med. *15*, 861–865.

Stahl-Hennig, C., Voss, G., Nick, S., Petry, H., Fuchs, D., Wachter, H., Coulibaly, C., Lüke, W., and Hunsmann, G. (1992). Immunization with tween-ether-treated SIV adsorbed onto aluminum hydroxide protects monkeys against experimental SIV infection. Virology *186*, 588–596.

Stahl-Hennig, C., Dittmer, U., Nisslein, T., Petry, H., Jurkiewicz, E., Fuchs, D., Wachter, H., Mätz-Rensing, K., Kuhn, E.M., Kaup, F.J., et al. (1996). Rapid development of vaccine protection in macaques by live-attenuated simian immunodeficiency virus. J. Gen. Virol. *77*, 2969–2981.

Stolte-Leeb, N., Sauermann, U., Norley, S., Fagrouch, Z., Heeney, J., Franz, M., Hunsmann, G., and Stahl-Hennig, C. (2006). Sustained conservation of CD4+ T cells in multiprotein triple modality-immunized rhesus macaques after intrarectal challenge with simian immunodeficiency virus. Viral Immunol *19*, 448–457.

Suh, Y.S., Park, K.S., Sauermann, U., Franz, M., Norley, S., Wilfingseder, D., Stoiber, H., Fagrouch, Z., Heeney, J., Hunsmann, G., et al. (2006). Reduction of viral loads by multigenic DNA priming and adenovirus boosting in the SIV-mac-macaque model. Vaccine *24*, 1811–1820.

Swigut, T., Alexander, L., Morgan, J., Lifson, J., Mansfield, K.G., Lang, S., Johnson, R.P., Skowronski, J., and Desrosiers, R. (2004). Impact of Nef-mediated downregulation of major histocompatibility complex class I on immune response to simian immunodeficiency virus. J. Virol. *78*, 13335–13344.

Utay, N.S., and Douek, D.C. (2016). Interferons and HIV infection: the good, the bad, and the ugly. Pathog. Immun. *1*, 107–116.

Wang, J.K., Kiyokawa, E., Verdin, E., and Trono, D. (2000). The Nef protein of HIV-1 associates with rafts and primes T cells for activation. Proc. Natl. Acad. Sci. USA *97*, 394–399.

Yu, H., Khalid, M., Heigele, A., Schmökel, J., Usmani, S.M., van der Merwe, J., Münch, J., Silvestri, G., and Kirchhoff, F. (2015). Lentiviral Nef proteins manipulate T cells in a subset-specific manner. J. Virol. *89*, 1986–2001.

Zhang, Y., Tada, T., Ozono, S., Yao, W., Tanaka, M., Yamaoka, S., Kishigami, S., Fujita, H., and Tokunaga, K. (2019). Membrane-associated RING-CH (MARCH) 1 and 2 are MARCH family members that inhibit HIV-1 infection. J. Biol. Chem. *294*, 3397–3405.

Zimin, A.V., Cornish, A.S., Maudhoo, M.D., Gibbs, R.M., Zhang, X., Pandey, S., Meehan, D.T., Wipfler, K., Bosinger, S.E., Johnson, Z.P., et al. (2014). A new rhesus macaque assembly and annotation for next-generation sequencing analyses. Biol. Direct *9*, 20.

STAR★METHODS

KEY RESOURCES TABLE

REAGENT or RESOURCE	SOURCE	IDENTIFIER
Antibodies		
Mouse monoclonal anti-HA tag (HA.C5)	Abcam	Cat# ab18181 RRID:AB_444303
Mouse monoclonal anti-HIV-1 p24 (39/5.4A)	Abcam	Cat# ab9071 RRID:AB_306981
Rabbit polyclonal anti-GFP	Abcam	Cat# ab290 RRID:AB_303395
Rabbit polyclonal anti-AU1 tag	Covance	Cat# MMS-130P RRID:AB_291308
Mouse monoclonal anti-β-actin	Abcam	Cat# ab8227 RRID:AB_306371
Rabbit polyclonal anti-GAPDH	Biolegend	Cat# 631401 RRID:AB_2247301
Mouse monoclonal anti-GAPDH	Santa Cruz	Cat# sc-365062 RRID:AB_10847862
Mouse monoclonal anti-CD4, APC conjugated	Thermo Fisher	Cat# MHCD0405 RRID:AB_10373698
Mouse monoclonal anti-HIV-1 core, FITC conjugated	Beckman Coulter	Cat# 6604665 RRID:AB_1575987
Mouse monoclonal anti-CD4, APC conjugated	Thermo Fisher	Cat# MHCD0405 RRID:AB_10373698
Mouse anti-human CD4 APC (clone RPA-T4)	BD Biosciences	Cat# 555349 RRID: AB_398593
Mouse anti-human CD28 PE (clone L293)	BD Biosciences	Cat# 348047 RRID: AB_400368
Mouse anti-human MHCI APC (clone W6/32)	BioLegend	Cat# 311410 RRID: AB_314879
Mouse anti-human, baboon, cynomolgus, rhesus CD3 V450 (clone SP34-2)	BD Biosciences	Cat# 560351 RRID: AB_1645168
Mouse anti-human CXCR4 APC (clone 12G5)	BD Biosciences	Cat# 555976 RRID: AB_398616
Mouse anti-human CXCR4 APC (clone 12G5)	BD Biosciences	Cat# 555976 RRID: AB_398616
Mouse anti-human, rhesus, cynomolgus, baboon MHCI PE (clone G46-2.6)	BD Biosciences	Cat# 555553 RRID: AB_395936
Mouse anti-human, baboon, cynomolgus, rhesus CD3 PE-Cy7 (clone SP34-2)	BD Biosciences	Cat# 557749 RRID: AB_396855
Mouse anti-human, baboon, cynomolgus, rhesus CD4 APC-H7 (clone L200)	BD Biosciences	Cat# 560837 RRID: AB_10563933
Mouse Anti-NHP CD45 (clone D058-1283)	BD Biosciences	Cat# 561489 RRID:AB_10683313
Mouse Anti-Human CD195 (clone 3A9, PE)	BD Biosciences	Cat# 556042 RRID:AB_396313
CD8 Mouse anti-Human (Clone: 3B5)	Invitrogen	Cat# 11320782 RRID:AB_1484754
CD8 antibody (RPA-T8)	BioLegend	Cat# 301024 RRID:AB_2561282
Anti-human CD20 Antibody (2H7)	BioLegend	Cat# 302301 RRID:AB_314249
Anti-human CD25 Antibody (M-A251)	BioLegend	Cat# 356101 RRID:AB_2561751
Anti-human HLA-DR Antibody (L243)	BioLegend	Cat# 307602 RRID:AB_314680
Mouse anti-human BST2 APC (clone RS38E)	BioLegend	Cat# 348410 RRID: AB_2067121
Anti-human CD69 Antibody (clone TP1.55.3)	Beckman Coulter	Cat# 6607110 RRID:AB_1575978
Bacterial and Virus Strains		
Escherichia coli XL-2 blue	Stratagene	Cat# 200150
XL2-Blue MRF' TM Ultracompetent cells	Agilent Technologies	Cat# 200151
Biological Samples		
Human: Peripheral blood mononuclear cells	DRK-Blutspende-dienst Baden-Württemberg, Ulm, Germany	N/A
Chemicals, Peptides, and Recombinant Proteins		
L-Glutamine	Pan Biotech	Cat# P04-80100
Penicillin-Streptomycin	ThermoFisher	Cat# 15140122
Biocoll separating solution	Biochrom	Cat# L6115
Lymphoprep	Stemcell	Cat# 07851

(*Continued on next page*)

Continued

REAGENT or RESOURCE	SOURCE	IDENTIFIER
Phorbol 12-myristate 13-acetate (PMA)	Sigma-Aldrich	Cat# 79346
Lipofectamine RNAiMAX Transfection Reagent	ThermoFisher	Cat# 13778150
TransIT®-LT1 Transfection Reagent	Mirus	Cat# MIR 2305
β-mercaptoethanol	Sigma Aldrich	Cat# M6250-100ML
HIV-1 p24 protein (ELISA standard)	Abcam	Cat# 43037
KPL SureBlue TMB Microwell Peroxidase Substrate	Medac	Cat# 52-00-04
4X Protein Sample Loading Buffer	LI-COR	Cat# 928-40004
2-Mercaptoethanol	Sigma-Aldrich	Cat# M6250-100ML
NucRed Live 647 ReadyProbes Reagent	ThermoFisher	Cat# R37106
Fixable Viability Dye eFluor 450	eBioscience	Cat# 65-0863-14
Critical Commercial Assays		
RosetteSep Human CD4+ T Cell Enrichment Cocktail	Stem Cell Technologies	Cat# 15062
EasySep Human Naive CD4+ T Cell Isolation Kit	Stemcell	Cat# 19555
QuikChange II XL Site-Directed Mutagenesis Kit	Agilent	Cat# 200522
Phusion High-Fidelity PCR Kit	ThermoFisher	Cat# F553L
DNA Ligation Kit Ver. 2.1	TaKaRa	Cat# 6022
GalScreen	Applied Bioscience	Cat# T1027
RNeasy Plus Mini Kit	QIAGEN	Cat# 74136
GAPDH Endogenous Control (VIC/TAMRA)	ThermoFisher	Cat# 4310884E
Luciferase Cell Culture Lysis 5X Reagent	Promega	Cat# E1531
Luciferase Assay System 10-pack	Promega	Cat# E1501
Deposited Data		
RNA-seq data	Gene Expression Omnibus (GEO) repository	Accession number GSE141626
Experimental Models: Cell Lines		
Human: HEK293T cells	ATCC	Cat# CRL-3216 RRID: CVCL_0063
Human: TZM-bl cells	NIH AIDS Reagent Program	Cat# 8129 RRID: CVCL_B478
P4 MAGI CCR5+ Cells	NIH AIDS Reagent Program	Cat# 3580
Jurkat NFAF-Luc cells	Fortin et al., 2004	N/A
Experimental Models: Organisms/Strains		
Rhesus macaques	German Primate Center	N/A
Recombinant DNA		
Plasmid: pCMV-VSV-G	Addgene	Cat# 8454
Plasmid: p99 PUR RPS EGFP	John L. Goodier	N/A
Plasmid: p99 JM111 EGFP	John L. Goodier	N/A
Plasmid: pL1RP-luc	Gerald Schumann	N/A
Plasmid: pBR322_SIVmac 239	F. Kirchhoff (Ulm University)	N/A
Plasmid: pCMV-VSV-G	Addgene	Cat# 8454
Software and Algorithms		
BD FACSDiva Version 8.0	BD Biosciences	https://www.bdbiosciences.com/en-us RRID: SCR_001456
Corel DRAW 2017	Corel Corporation	https://www.coreldraw.com/en/
GraphPad Prism Version 7	GraphPad Software, Inc.	https://www.graphpad.com RRID: SCR_002798
ImageJ	Open source	https://imagej.nih.gov/ij/
LI-COR Image Studio Lite Version 5.0	LI-COR	https://www.licor.com/ RRID : SCR_013715

LEAD CONTACT AND MATERIALS AVAILABILITY

Further information and requests for resources and reagents should be directed to and will be fulfilled by the Lead Contact, Frank Kirchhoff (frank.kirchhoff@uni-ulm.de). All unique/stable reagents generated in this study are available from the Lead Contact without restriction.

EXPERIMENTAL MODEL AND SUBJECT DETAILS

Ethical statement for human samples

Experiments involving human blood and CD4+ T cells were reviewed and approved by the Institutional Review Board (i.e., the Ethics Committee of Ulm University). Individuals and/or their legal guardians provided written informed consent prior to donating blood. All human-derived samples were anonymized before use. The use of established cell lines (HEK293T, Jukat and P4-CCR5 cells) did not require the approval of the Institutional Review Board.

Ethical statement for animal experiments

Nine male and three female purpose-bred rhesus macaques (*Macaca mulatta*) that were 4.5-5.5 years old when allocated to the experiment were obtained from the German Primate Center (DPZ) breeding colony. Animals from this study were cared for by qualified staff of the DPZ and housed according to the German Animal Welfare Act which complies with the European Union guidelines on the use of non-human primates for biomedical research and the Weatherall report. The study was approved by the Lower Saxony State Office for Consumer Protection and Food Safety and performed with the project licenses 33.19-42502-04-12/0758 and 33.19-42502-04-15/2001. The DPZ has the permission to breed and house non-human primates under license number 392001/7 granted by the local veterinary office and conforming with § 11 of the German Animal Welfare act. Socially compatible monkeys were housed in groups of two by combining cages. If animals had to be caged individually they had constant visual, olfactory and acoustic contact to their roommates and could still groom their neighbors through small mash inserts in the separating side walls. Each cage was equipped with a perch. The animals had water access *ad libitum* and were fed with dry monkey biscuits containing adequate carbohydrate, energy, fat, fiber (10%), mineral, protein, and vitamin content twice daily. The feed was supplemented by fresh fruit or vegetables and other edible objects like nuts, cereal pulp and different seeds to offer variety to the diet. Moreover, for environmental enrichment monkeys were provided feeding puzzles boards, alternate toys and wood sticks for gnawing. During the study, animals were assessed by experienced keepers twice a day for any signs of distress, pain or sickness by checking water and feed intake, feces consistency and the general condition. In case of any abnormal presentation, animals were attended by veterinarians.

Cell lines

Human Embryonic Kidney (HEK) 293T cells (DuBridge et al., 1987) were obtained from the American Type Culture Collection (ATCC). P4-CCR5 reporter cells were kindly provided through the NIH AIDS Reagent Program. Both cell lines were cultured in Dulbecco's Modified Eagle Medium (DMEM) supplemented with 10% heat-inactivated fetal calf serum (FCS), 2 mM L-glutamine, 100 units/ml penicillin and 100 μg/ml streptomycin. P4-CCR5 cells express CD4, CCR5 and CXCR4 and contain the β-galactosidase genes under the control of the HIV-1 promoter (Charneau et al., 1994). Jurkat-NFAT cells contain a luciferase gene under the control of the NFAT promoter (Fortin et al., 2004) and were cultured in RPMI-1640 medium supplemented with 10% heat-inactivated FCS, 2 mM L-glutamine, 100 units/ml penicillin, 120 μg/ml streptomycin and 200 μg/ml G418 (geneticin). Jurkat-NFAT cells were transduced with eGFP-reporter proviral constructs and stimulated with 4 μg/ml PHA for 16 hr before lysis and determination of luciferase activity.

Primary cell culture

Peripheral blood mononuclear cells (PBMCs) from healthy human donors were isolated using lymphocyte separation medium (Biocoll separating solution; Biochrom), stimulated for 3 days with phytohemagglutinin (PHA; 2 μg/ml), and cultured in RPMI 1640 medium with 10% fetal calf serum and 10 ng/ml interleukin-2 (IL-2) prior to infection or first infected and then cultured in medium only until stimulation with PHA at day 3 or 6. CD4+ T cells from healthy human donors were isolated using RosetteSep Human CD4+ T Cell Enrichment Cocktail (Stemcell) and lymphocyte separation medium (Biocoll separating solution; Biochrom), stimulated for 3 days with PHA (2 μg/ml), and cultured in RPMI 1640 medium with 10% FCS and 10 ng/ml interleukin-2 (IL-2) prior to infection.

METHOD DETAILS

Proviral constructs

Proviral HIV-1 NL4-3-IRES-eGFP constructs containing an internal ribosome entry site (IRES) and encoding the enhanced version of the green fluorescent protein (eGFP) were generated as described (Schindler et al., 2003). Overlap-extension PCR was used to replace the NL4-3 *nef* in wt or IRES-eGFP HIV-1 constructs by wt or mutant SIV_{mac239} *nef* genes as described (Schindler et al.,

2006). The integrity of all PCR-derived inserts was confirmed by sequencing. The control HIV-1 NL4-3-IRES-eGFP constructs expressing the NL4-3, NA7, and SIV_{mac239} Nefs or containing a deleted (*Δnef*) or disrupted (*nef**) *nef* gene have been reported previously (Münch et al., 2007).

Expression vectors

Cloning of *nef* alleles into the bi-cistronic CMV promoter-based pCG expression vector co-expressing the enhanced green fluorescent protein (eGFP) was described previously (Schindler et al., 2006). In brief, *nef* genes were amplified by PCR with flanking primers introducing a C-terminal AU1 tag and XbaI and MluI restriction sites for cloning into the pCG IRES eGFP vector.

Western blot

To examine the expression of primate lentiviral Nef proteins, HEK293T cells were transfected in 6-well dishes with 5 μg DNA of pCG IRES eGFP vectors expressing AU1-tagged Nefs. Two days post-transfection, cells were lysed with Co-IP buffer (150 mM NaCl, 50 mM HEPES, 5 mM EDTA, 0.1% NP40, 0.5 mM sodium orthovanadate, 0.5 mM NaF, protease inhibitor cocktail from Roche, pH 7.5) and reduced in the presence of β-mercaptoethanol by boiling at 95°C for 10 min. Proteins were separated in 4 to 12% Bis-Tris gradient acrylamide gels (Invitrogen), blotted onto polyvinylidene difluoride (PVDF) membrane, and incubated with anti-AU1 (MMS-130P; Covance), anti-GFP (ab290; Abcam), and anti-β-actin (ab8227; Abcam) antibodies.

Flow cytometry

To analyze Nef function, CD4, MHC-I, CD28, CD3, CXCR4 and tetherin surface levels in PBMCs or CD4+ T cells transduced with HIV-1 NL4-3 constructs coexpressing Nef and GFP or containing a disrupted *nef* gene as control were measured as described previously (Sauter et al., 2009; Schindler et al., 2006). Briefly, the cells were washed and stained with fluorescently labeled antibodies. Staining was performed for 30 to 60 min at 4°C. After fixing the cells with 2% PFA, measurement was performed using a BD FACSCanto II. For flow cytometry, the following antibodies were titrated and validated using matching isotype controls from the same supplier: anti-human CD3 (1:40, SP34-2, BD, Cat# 557749 or 1:5, UCHT1, BD, Cat# 555335), anti-human CD4 (1:20, L200, BD, Cat# 560811 or 1:5, RPA-T4, BD, Cat# 555349), anti-human MHC-I (1:20, G46-2.6, BD, Cat# 561346), anti-human CXCR4 (1:40, 12G5, BD, Cat# 560670), anti-human CD28 (1:10, CD28.2, BD, Cat# 559770 or 1:20, CD28.2, BD, Cat# 560684) and anti-human tetherin (1:20, RS38E, BioLegend, Cat# 348410).

For analysis of rhesus macaque cells, 50 μL of whole blood were stained for 30 min at RT in the dark with different mixtures of monoclonal antibodies (mAb) directed against CD3 (clone SP34-2, Alexa Fluor 700), CD4 (clone L200; Horizon V450), CD45 (clone D058-1283, Horizon V500), CD80 (clone L307.4, PE) and CD195 (clone 3A9, PE), all from BD Biosciences, Heidelberg, Germany; CD8 (clone 3B5, Pacific Orange, Invitrogen, Carlsbad, USA, due to adjustment in the staining protocol replaced by CD8 (clone RPA-T8, PerCP-Cy5.5; for animals 2729, 2746, 15899, 15925, 15926), CD20 (clone 2H7, PE-Cy7), CD25 (clone M-A251, APC-Cy7, replaced by APC-Fire for above mentioned animals), HLA-DR (clone L243, APC-Cy7) all from BioLegend, San Diego, USA as well as CD69 (clone TP1.55.3, ECD) Beckman Coulter, Krefeld, Germany. Lysis of residual RBCs (red blood cells) and fixation was performed by subsequent incubation with 1 mL RBC lysis/fixation solution from BioLegend for 15 min. Following a washing step with staining buffer (phosphate-buffered saline with 5% bovine serum albumin) cells were acquired using a LSRII cytometer (BD Biosciences). Data analysis was performed using FlowJo 9.6.4 (Treestar, Ashland, OR, USA).

Virus stocks and transductions

To generate virus stocks, HEK293T cells were co-transfected with the proviral HIV-1 or SIVmac constructs alone (for assays on infectivity and replication) or together with a plasmid (pHIT-G) expressing the vesicular stomatitis virus G protein (VSV-G) to achieve comparably high initial infection levels for flow-cytometric analyses. Two days post-transfection, supernatants containing infectious virus was harvested. The amount of HIV-1 capsid protein was quantified by p24 antigen ELISA as described (Krapp et al., 2016) for normalization of the virus dose. CD4+ T cells, PBMCs or Jurkat cells were transduced with VSV-G pseudotyped virus stocks containing normalized quantities of p24 antigen. Four hours post-transduction, cells were washed three times in PBS, resuspended in RPMI 1640 medium with 10% heat-inactivated FCS, 2 mM L-glutamine and antibiotics and cultured for two to three days prior to flow cytometric analysis.

Viral infectivity

Virus infectivity was determined using P4-CCR5 as described (Münch et al., 2007). Briefly, the cells were sown out in 96-well dishes in a volume of 100 μL and infected by overnight incubation with virus stocks, containing 1 ng of p24 antigen, produced by transfected HEK293T cells. Three days post-infection, viral infectivity was detected using the Gal-Screen kit from Applied Biosystems as recommended by the manufacturer. β-galactosidase activity was quantified as relative light units per second using the Orion microplate luminometer.

NF-AT assay

Jurkat cells stably transfected with an NF-AT-dependent reporter gene vector (Fortin et al., 2004) were either left uninfected or transduced with HIV-1 IRES eGFP constructs expressing various *nef* alleles. Except for those cells used as controls, cultures

were treated with PHA (4 μg/ml; Murex). Luciferase activity was measured and n-fold induction determined by calculating the ratio of measured relative light units (RLU) of treated samples to that of untreated samples, as described previously (Khalid et al., 2012).

Experimental animals, virus inoculation and specimen collection

Twelve purpose-bred young adult rhesus macaque, 9 males and 3 females, were obtained from the DPZ breeding colony. When allocated to the experiment, they were 4.5-5.5 years old with a bodyweight between 4.7-8.9 kg. All monkeys were seronegative for Simian Retrovirus Typ D, Simian Immunodeficiency Virus, and Simian T-lymphotropic Virus. Animals were randomly distributed to two groups with six monkeys each. For blood collection from the femoral vein by using the vacutainer system (BD), animals were anesthetized by intramuscular (i.m.) injection of a mixture of 5 mg ketamine, 1 mg xylazine, and 0.01 mg atropine per kilogram body weight (BW). For virus inoculation, medical interventions like removal of peripheral lymph nodes, and as premedication for euthanasia, all for which a deeper anesthesia was required, animals were administered i.m. a mixture of 5-10 mg ketamine, 1-2 mg xylazine, and 0.01-0.02 mg atropine per kg BW. At necropsy, animals were euthanized by an overdose of 160-240 mg sodium pentobarbital per kg BW injected into the circulation. Four males and two females were inoculated intravenously with 1 mL of cell-free culture supernatant containing 500 median tissue culture infectious doses of CD3ko-Nef SIV_{mac239}. The animals of the other group (five males and one female) received SIV_{mac239} wild-type at the same dose and by the same route as for the mutant virus. Blood samples were collected four to five times before infection, two to three times up to day 7, thereafter at weeks 2, 3, and 4, followed by 2-4-week intervals until 24 weeks post infection (wpi) and thereafter around every 8 weeks until euthanasia because of the development of AIDS-like disease or the end of the study (weeks 82-89). Peripheral lymph nodes were surgically removed once or twice before infection and up to six times between 2 and 66 wpi.

RNA Purification

Total RNA extraction was performed using the Paxgene RNA purification kit (QIAGEN, Hilden, Germany). Briefly, 2.5 ml of whole blood was collected in Paxgene blood RNA tubes and total RNA extraction was performed using the Paxgene RNA purification kit according to manufacturer's specifications; on-column DNase digestion was also performed to remove Genomic DNA. RNA integrity of the extracted RNA was assessed by Agilent Bioanalyzer (Agilent Technologies, Santa Clara, CA, USA) capillary electrophoresis on a Bioanlayzer RNA nanochip.

Viral RNA and cell-associated viral load quantitation

Viral RNA was extracted from 200 μl plasma using QiaAmp viral RNA isolation mini kit. For qRT-PCR of viral RNA, 8.5 μl RNA-solution were reverse transcribed and amplified using TaKaRa Prime-Script-One-Step-RT-PCR kit (TaKaRa Bio Europe) using primers *gag* forward (5′-ACCCAGTACAACAAATAGGTGGTAACT-3′), *gag* reverse (5′-TCAATTTTACCCAG-GCATTTAATGT-3′) and a fluorescent probe (5′-6FAM(6-carboxyfluorescein)-TGTCCACCT-GCCATTAAGCCCGAG-TAMRA(6-carboxytetramethylrhodamine-3′). Reverse transcription was performed at 45°C for 5 min, amplification was started by an initial denaturation step at 95°C for 10 s, followed by 45 cycles of denaturation for 5 s at 95°C and annealing and elongation at 60°C for 30 s using the Rotor-Gene Q apparatus and software (QIAGEN).

The cell-associated virus load in peripheral blood was determined by a limiting dilution co-cultivation assay as described (Stahl-Hennig et al., 1992, 1996). Instead of testing supernatants of cell cultures for SIV p27, SIV positive cultures were identified by immunoperoxidase staining for intracellular viral proteins with a SIV-hyperimmune serum from an infected macaque as reported (Norley et al., 1993). To increase adherence of indicator cells, plates used for staining were coated with Concanavalin A (Gundlach et al., 1998).

ELISpot assay

IFN-γ ELISpots were performed to determine the SIV-specific T cell response in the infected monkeys (Suh et al., 2006). For virus-specific stimulation, SIV Gag (EVA7066.1-72), Nef (EVA777) and Tat (EVA7069) peptides, and whole SIVmac251 particles chemically inactivated with aldrithiol-2 (ARP1018.1&2, SIV AT-2) were used essentially as described (Sauermann et al., 2017). All reagents for the T cell ELISpot assay were kindly provided by the Centre for AIDS Reagents (National Institute for Biological Standards and Control), UK.

B cell ELISpot

For the detection of antibody-secreting memory (ASC) B cells PBMCs were seeded in 48-well plates (Greiner Bio-One) at a concentration of $2x10^6$/ml in RPMI1640 supplemented with 10% fetal calf serum and 1% penicillin and 1% streptomycin (complete RPMI) and pre-activated with 10 μg/ml pokeweed mitogen (Sigma), and 5 μg/ml CpG (Cytosin-Phosphat-Guanin) class B ODN 2006 (InvivoGen) for three days. For the B cell ELISpot a commercial kit from Mabtech was used. In short, MultiScreen plates with the Immobilon-P PVDF (Polyvinylidenfluorid) membrane (MSIPS4510, Merck Millipore) were activated with 35% ethanol, and washed five times with distilled water. For the detection of total IgG secreting cells, membranes were coated with an IgG capture antibody (MTl91/145) and for the detection of memory B cells with recombinant SIVgp130 (EVA670, NIBSC). Plates coated with bovine serum albumin (PAN-Biotech) served as negative controls. All plates were incubated at 4°C overnight, subsequently washed with PBS and incubated with complete RPMI for 3 h at 37°C. For detection of total IgG secreting cells 100,000 freshly isolated PBMC were added

per well. For detection of memory cells, PBMC stimulated with SIV antigen or kept in the negative control plates were added at a concentration of 200,000 cells per well. Plates were incubated for 20 h at 37°C. Thereafter, plates were washed five times with PBS. Antibodies were detected using biotin-conjugated anti human IgG MT78/145 followed by incubation with streptavidin-alkaline-phosphatase and staining with BCIP/NBT (bromo-chloro-indolyl phosphate/nitro blue tetrazolium chloride) PLUS (Moss) according to standard protocols. For enumeration of spots a Bioreader 6000-V (BIOSYS) was used. To calculate the SIV-specific ASC, total IgG wells were used as positive control after subtracting spots measured in the negative control wells. Memory B cells were defined as the number of ASC after subtraction of spots from the negative controls and expressed as percent of total IgG secreting ASC per $1x10^6$ PBMC.

Serological testing

To analyze for the SIV-specific humoral immune response in blood, a standard ELISA was performed using recombinant SIV p27 (EVA643.2) and SIV gp130 (cat. # 100841) kindly provided by the Centre for AIDS Reagents (National Institute for Biological Standards and Control), UK. Antigens were coated at 50 ng protein per well. Antibody levels were assessed using 1:200-diluted plasma (Stolte-Leeb et al., 2006).

MHC typing

Animals were negative for the major histocompatibility complex (MHC) class I Mamu-A1*001:01, and -B*017:01 alleles known to be associated with slow disease progression. Typing was performed as described previously (Sauermann et al., 2008).

Transcriptome analysis

The collection of blood peripheral blood mononuclear cells (PBMC) and peripheral lymph node (LN) samples from wt SIV_{mac239} and CD3ko-Nef infected animals was carried out in two sets. Set A consists of PBMC and LN samples from wt (n = 3) and CD3ko-Nef (n = 3) infected animals at only post-infection time points. There were no LN samples collected at 2 wpi in Set A. In addition to PBMC samples from infected animals, PBMC samples in Set A were also collected from uninfected control animals (n = 10). Set B consists of PBMC and LN samples from wt (n = 3) and CD3ko-Nef (n = 3) infected animals before and after infection.

RNA was extracted from these samples using RNeasy Mini kits (QIAGEN, CA) with DNase digest and QIAcube automation stations. Extracted RNA was quantified using a NanoDrop 2000 spectrophotometer (Thermo Scientific Inc. Wilmington, DE) and the quality was assessed by Bioanalyzer analysis (Agilent Technologies, Santa Clara, CA). For Set A samples, libraries were prepared using the Illumina (Illumina Inc. San Diego, Ca, USA) TruSeq mRNA stranded kit, as per manufacturer's instructions, with 400 ng of total RNA as input. For Set B samples, 10 ng of total RNA was used as input for cDNA amplification using 5′ template-switch PCR with the Clontech SMART-Seq v4 Ultra Low Input RNA kit. Further, for this set, amplified cDNA was fragmented and appended with dual indexed bar codes using Illumina NexteraXT DNA Library Prep kits. The amplified libraries from both sets were validated by capillary electrophoresis on the Agilent 4200 TapeStation. The libraries were normalized, pooled and sequenced on the Illumina HiSeq 3000 system employing a single-end 101 cycles run at average read depths of 16 million reads/sample (Set A) and 25 million reads/sample (Set B).

For further analysis, Set A and B samples were combined. The sequencing data was demultiplexed using Illumina bcl2fastq version 2.20.0.422. The quality of raw reads was assessed with FastQC version 0.11.8 (http://www.bioinformatics.babraham.ac.uk/projects/fastqc). Reads were mapped to the MacaM version 7 assembly of the Indian rhesus macaque genomic reference (Zimin et al., 2014) assembly available at: https://www.unmc.edu/rhesusgenechip/index.htm RhesusGenome using STAR version 2.5.2b with default alignment parameters (Dobin et al., 2013). Abundance estimation of raw read counts per transcript was done internally with STAR using the htseq-count algorithm (Anders et al., 2015).

DESeq2 version 1.22.1 R package was used to produce normalized read counts and a regularized log expression table (Love et al., 2014). Here, RNA-Seq data analysis was performed separately for the PBMC and LN datasets. Further, DESeq2 was used to compute the differential expression estimation between infected and uninfected samples as well as CD3ko versus wt comparisons at 2 wpi (peak-viremia). PBMC and LN infected datasets consist of samples collected at 2 wpi from wt SIVmac239 and CD3ko-Nef infected animals. PBMC uninfected dataset consists of blood samples collected from control animals and those from infected animals at 4 weeks before infection. LN uninfected dataset consists of samples collected from infected animals at 2 weeks before infection.

Gene set enrichment analysis and heatmaps

Gene set enrichment analysis (GSEA) was performed on the PBMC and LN regularized log expression data using the Broad Institute GSEA desktop module (https://www.broadinstitute.org/gsea/). Enrichment analysis was performed for CD3ko-Nef versus uninfected, wt versus uninfected and CD3ko-Nef versus wt datasets for PBMC and lymph node samples. Heatmaps were generated to visualize the expression patterns of the leading edge genes in the indicated gene sets for that enrichment analysis. For both the PBMC and LN regularized log expression data, the expression for each gene was normalized by the mean expression across all samples (CD3ko-Nef, wt and uninfected samples). These were then plotted based on a gradient color-scale. The gene sets have been provided in the Table S1.

Multiplex cytokine profile analysis

To simultaneously detect various chemokines and cytokines, we performed a multiplex assay as previously described (Giavedoni, 2005). In brief, we applied the Luminex technology that utilizes combinations of two monoclonal antibodies to identify specific targets; one that captures the target molecule and a second coupled to specific beads directed to a different epitope in the same target, to detect the captured analyte. Subsequent analyses by flow cytometry allows to discriminate between particles on the basis of color and size, enabling simultaneous detection of numerous molecules. We have previously identified reagents that recognize the rhesus macaque homologs of human molecules and allow the identification of multiple monkey cytokines and chemokines in a single test tube (Giavedoni, 2005; Pantoja et al., 2017). A total of 15 cytokines/chemokines were measured in macaque plasma by Luminex at baseline, days 5, 7, and 10, and weeks 2, 3 4, and 8 post SIV infection. The analytes included: B cell activating factor (BAFF), eotaxin (CCL11), interferon alpha (IFN-α) and gamma (IFN-γ), interleukin-6 (IL-6), IL-8, and IL-12 p40, IL-1 receptor antagonist (IL-1Ra), monokine induced by IFN-gamma (MIG/CXCL9), macrophage inflammatory protein 1-alpha (MIP-1α/CCL3) and beta (MIP-1β/CCL4), C-X-C motif chemokine 10 (CXCL10/IP-10) and 11 (CXCL11/I-TAC), perforin, and tumor necrosis factor-alpha (TNF-α). Only analytes quantifiable above the limit of detection are presented.

QUANTIFICATION AND STATISTICAL ANALYSIS

Statistical analyses were performed with GraphPad PRISM (GraphPad Software) and Microsoft Excel. P values were calculated using the two-tailed unpaired Student's-t test. Correlations were calculated with the linear regression module. Unless otherwise stated, all experiments were performed three times and the data are shown as mean $\pm$ SEM. Significant differences are indicated as: *$p < 0.05$; **$p < 0.01$; ***$p < 0.001$. Statistical parameters are specified in the figure legends.

DATA AND CODE AVAILABILITY

RNA-Seq data is available in the Gene Expression Omnibus (GEO) repository under accession number GEO: GSE141626.

Cell Reports, Volume *30*

Supplemental Information

Nef-Mediated CD3-TCR Downmodulation Dampens Acute Inflammation and Promotes SIV Immune Evasion

Simone Joas, Ulrike Sauermann, Berit Roshani, Antonina Klippert, Maria Daskalaki, Kerstin Mätz-Rensing, Nicole Stolte-Leeb, Anke Heigele, Gregory K. Tharp, Prachi Mehrotra Gupta, Sydney Nelson, Steven Bosinger, Laura Parodi, Luis Giavedoni, Guido Silvestri, Daniel Sauter, Christiane Stahl-Hennig, and Frank Kirchhoff

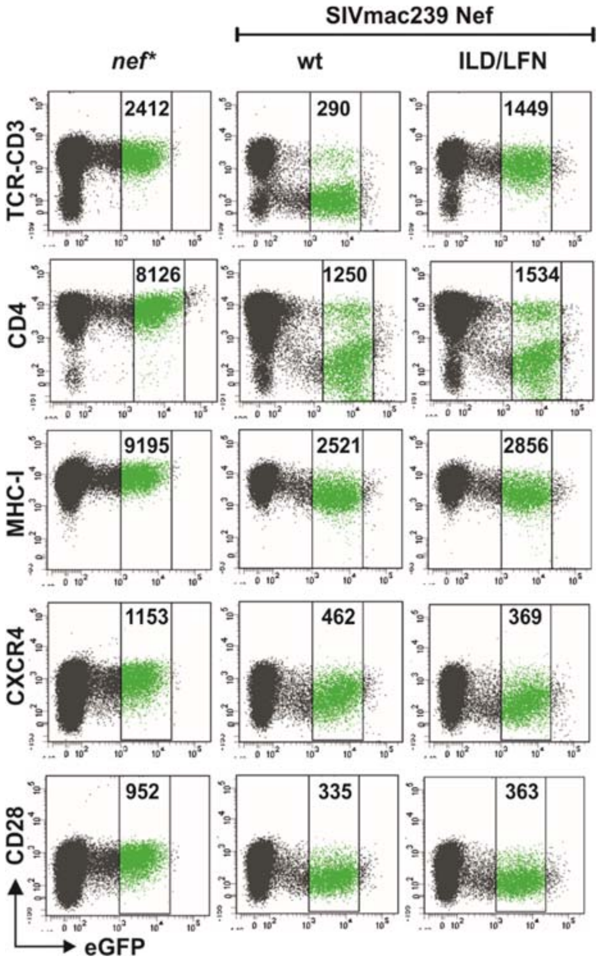

Figure S1 (related to Figure 1). Modulation of CD3, CD4, MHC-I, CXCR4 and CD28 by SIV$_{mac239}$ wt and CD3ko (ILD/LFN) Nef proteins. Human PBMCs were stimulated with PHA for three days and subsequently transduced with VSV-G-pseudotyped HIV-1 NL4-3 IRES-eGFP constructs containing a defective *nef* gene (*nef**) or the wt or mutant SIV$_{mac239}$ *nef* alleles. Surface expression was analyzed by flow cytometric analysis 2 days later.

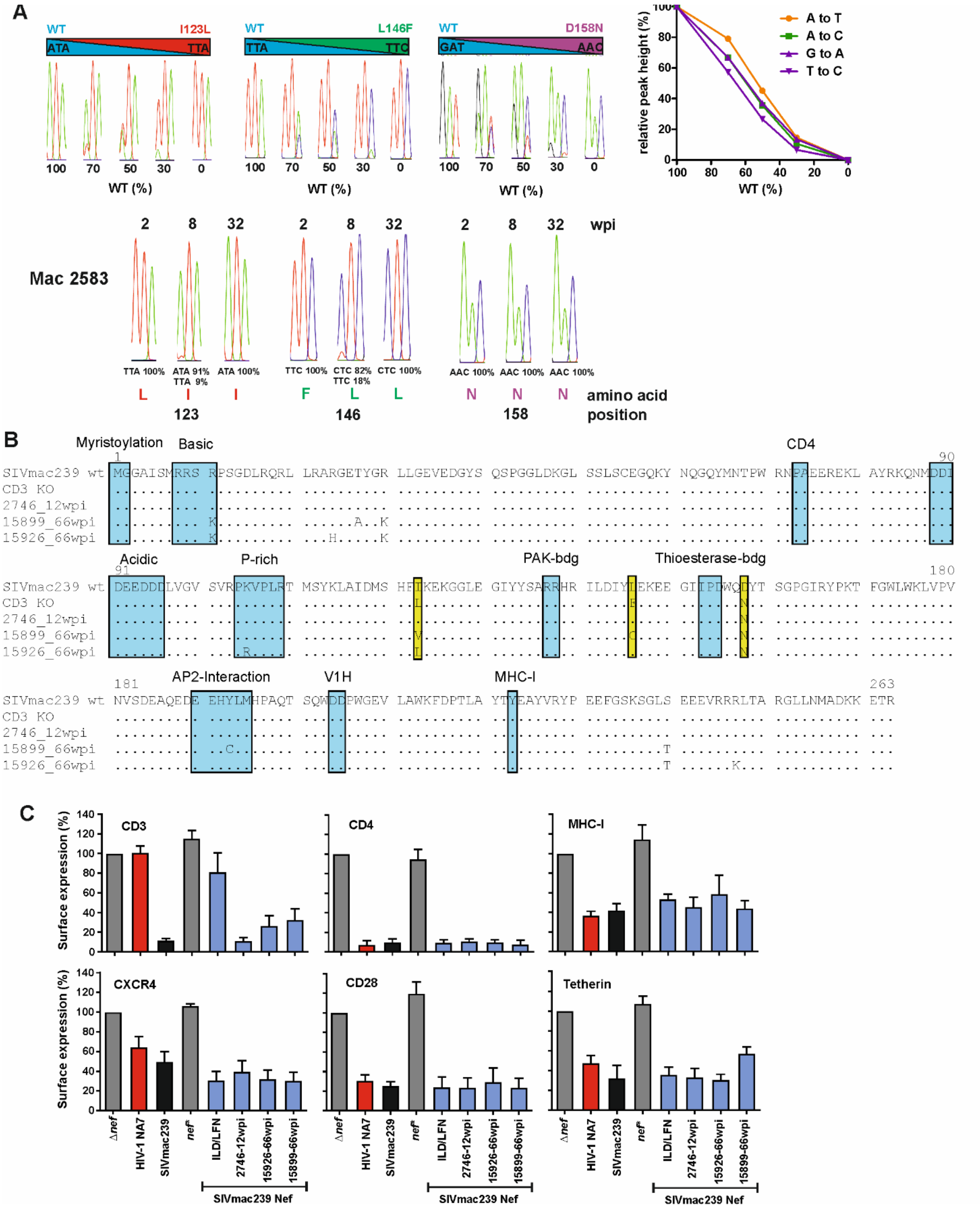
A
WT
I123L
ATA
TTA
WT
L146F
TTA
TTC
WT
D158N
GAT
AAC
100 70 50 30 0
WT (%)
relative peak height (%)
A to T
A to C
G to A
T to C
2 8 32 wpi
Mac 2583
TTA 100%
ATA 91% TTA 9%
ATA 100%
TTC 100%
CTC 82% TTC 18%
CTC 100%
AAC 100%
AAC 100%
AAC 100%
L I I F L L N N N
123
146
158
amino acid position
B
Myristoylation
Basic
CD4
Acidic
P-rich
PAK-bdg
Thioesterase-bdg
AP2-Interaction
V1H
MHC-I
SIVmac239 wt
CD3 KO
2746_12wpi
15899_66wpi
15926_66wpi
SIVmac239 wt MGGAISMRRS RPSGDLRQRL LRARGETYGR LLGEVEDGYS QSPGGLDKGL SSLSCEGQKY NQGQYMNTPW RNPAEEREKL AYRKQNMDDI
SIVmac239 wt DEEDDDLVGV SVRPKVPLRT MSYKLAIDMS HFIKEKGGLE GIYYSARRHR ILDIYLEKEE GIIPDWQDYT SGPGIRYPKT FGWLWKLVPV
SIVmac239 wt NVSDEAQEDE EHYLMHPAQT SQWDDPWGEV LAWKFDPTLA YTYEAYVRYP EEFGSKSGLS EEEVRRRLTA RGLLNMADKK ETR
C
Surface expression (%)
CD3
CD4
MHC-I
CXCR4
CD28
Tetherin
Δnef
HIV-1 NA7
SIVmac239
nef*
ILD/LFN
2746-12wpi
15926-66wpi
15899-66wpi
SIVmac239 Nef

Figure S2 (related to Figure 2). Detection and functional analysis of reversions detected in rhesus macaques infected with the CD3ko-Nef SIV$_{mac239}$ construct. (**A**) Quantitative detection of reversions in the CD3ko-Nef. Upper, PCR amplification products of wt and *nef* mutant SIV$_{mac239}$ constructs were mixed at the indicated molar ratios and sequenced as described in the methods section. The right panel shows the percentage of peak fluorescence for wt nucleotides compared with the total fluorescence. Lower, RNA was isolated from plasma of animal 2583 at the indicated weeks post-infection, the *nef* coding region amplified by RT-PCR and sequenced directly. The percentage of reversions was calculated from the standard curves shown above. (**B**) Alignment of the wild-type and CD3ko-Nef SIV$_{mac239}$ Nef amino sequences with those detected in animals 2746, 15899 and 15296 at the indicated weeks post-infection. Dots represent amino acid identity; known functional domains are indicated. (**C**) Human PBMCs were transduced with VSV-G-pseudotyped HIV-1 NL4-3 IRES-eGFP constructs expressing the indicated *nef* alleles and assayed for surface expression of CD3, CD4, MHC-I, CXCR4 and CD28. Shown are average values ± SD obtained for three different donors. To measure the effect on tetherin, HEK293T cells were cotransfected with a pCG vector expressing macaque tetherin and an NL4-3 proviral construct expressing the indicated *nef* alleles. Two days later, cell surface expression of tetherin was determined by FACS.

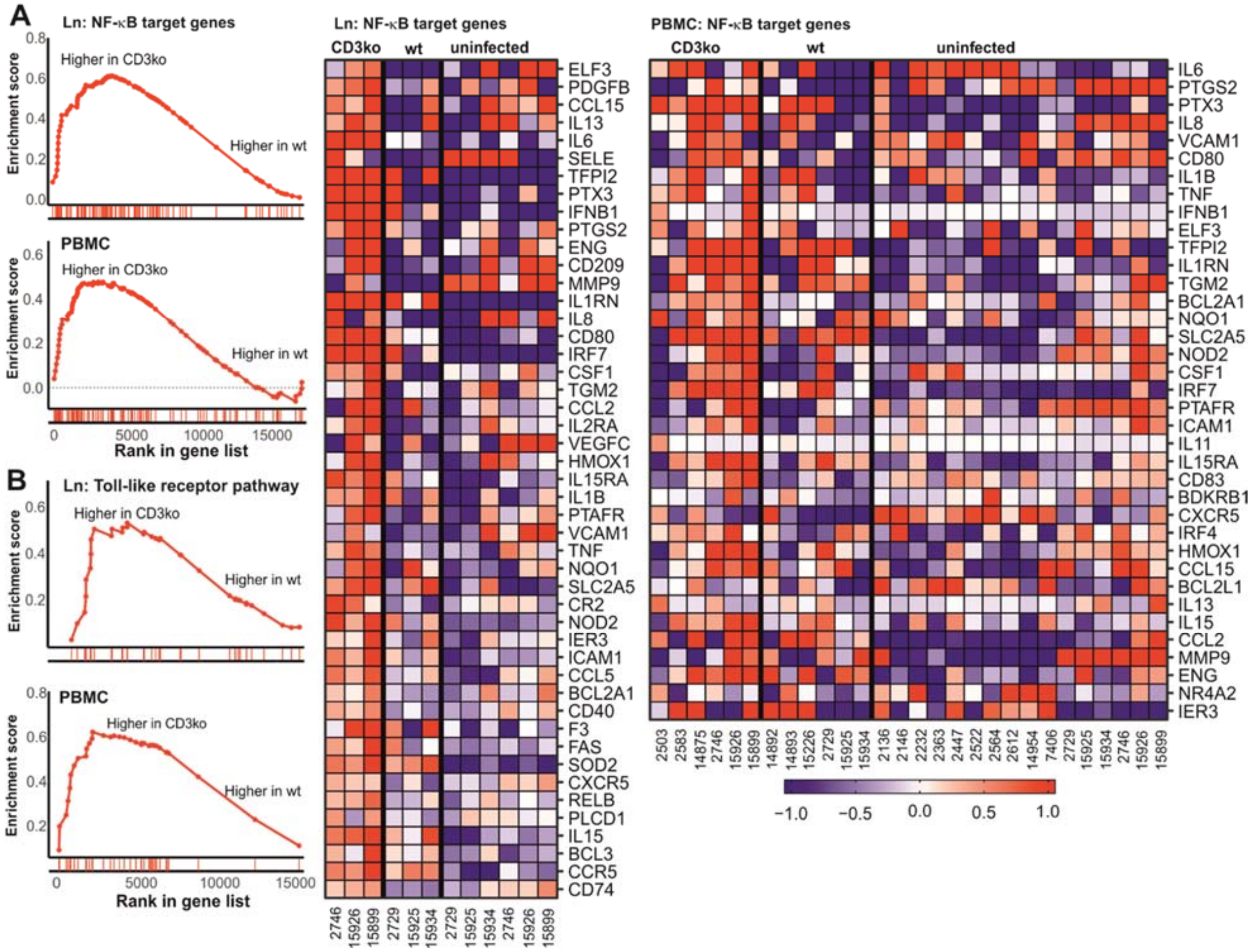

Figure S3 (related to Figure 3). Lack of Nef-mediated CD3 downmodulation affects expression of genes involved in NF-κB and TLR signaling. (**A, B**) GSEA plots and heat maps of CD3ko-Nef vs wt in lymph node and PBMC datasets for (A) NF-κB target gene sets and (B) the TLR signaling pathway gene set. The CD3ko-Nef and wt datasets consist of samples collected at 2 wpi from CD3ko-Nef and wt SIV_{mac239} infected animals, respectively. The running enrichment score (y-axis) is indicated for each gene ordered by their rank in the whole data set for that specific comparison (shown by the bars below the x-axis). The right panels show heat maps for the leading-edge genes of the NF-κB target gene set for PBMC and lymph node samples of the CD3ko-Nef and wt at 2 wpi, and uninfected datasets. The color scale is shown at the bottom, with lowest to highest gene expression across all animals represented by the blue to red color gradient

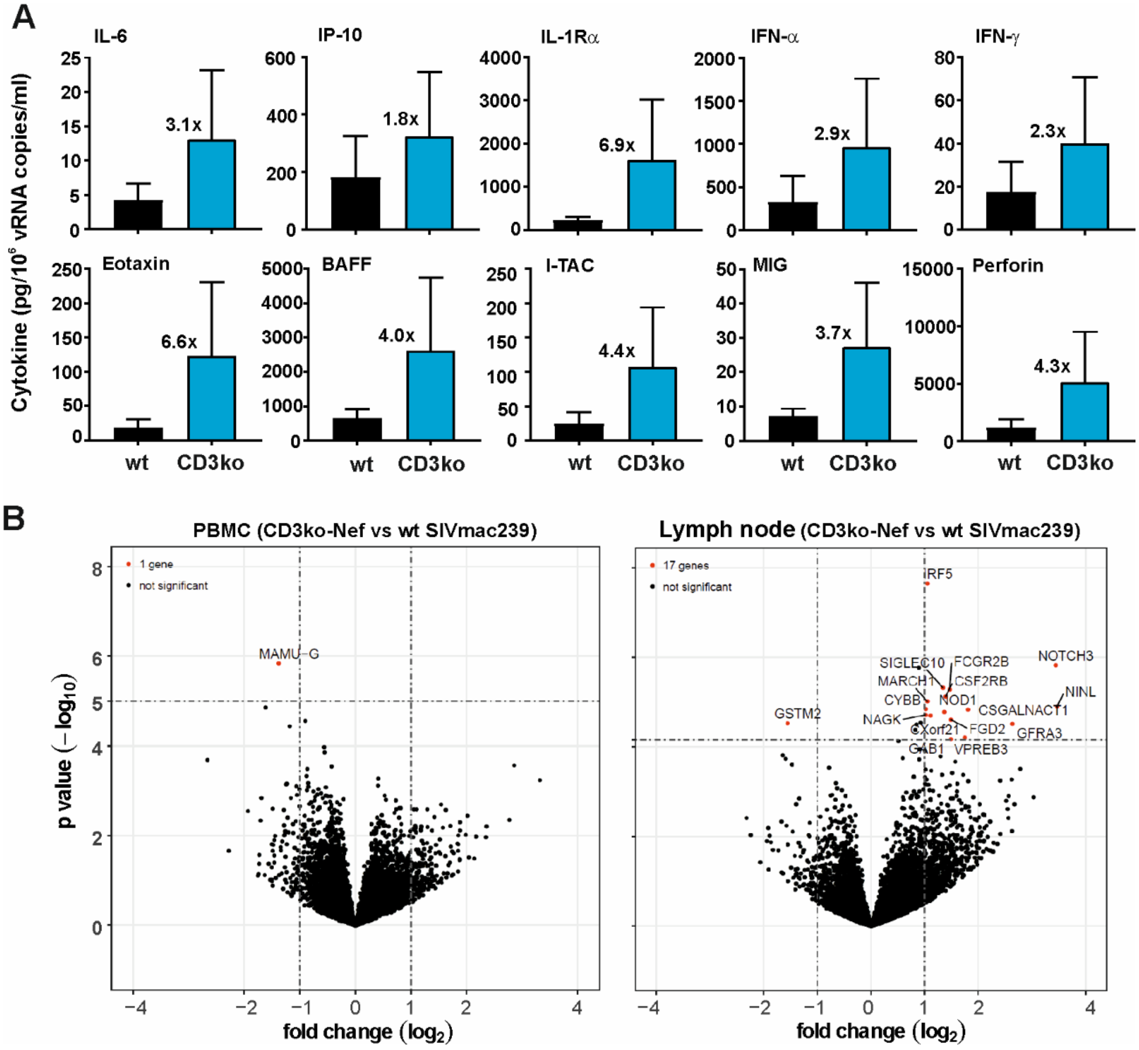

Figure S4 (related to Figure 4). Proinflammatory cytokines and differential gene expression in wt and CD3ko-Nef SIV$_{mac239}$ infection. (**A**) Levels of proinflammatory cytokines in plasma of the 12 rhesus macaques infected with wt or CD3ko-Nef SIV$_{mac239}$ (mean ±SEM) were measured by Luminex. Shown are the quantities of cytokines detected at peak viremia (2 wpi) normalized to the RNA levels of the individual animals. The numbers next to the blue bars indicate n-fold difference. (**B**) Volcano plots depicting the fold-change (CD3ko-Nef vs wt SIV mac239 at 2 wpi) and statistical significance for genes from (left) PBMC and (right) lymph nodes of rhesus macaques. The red dots highlight the differentially expressed genes showing >2-fold change in their expression, with an FDR <0.05.

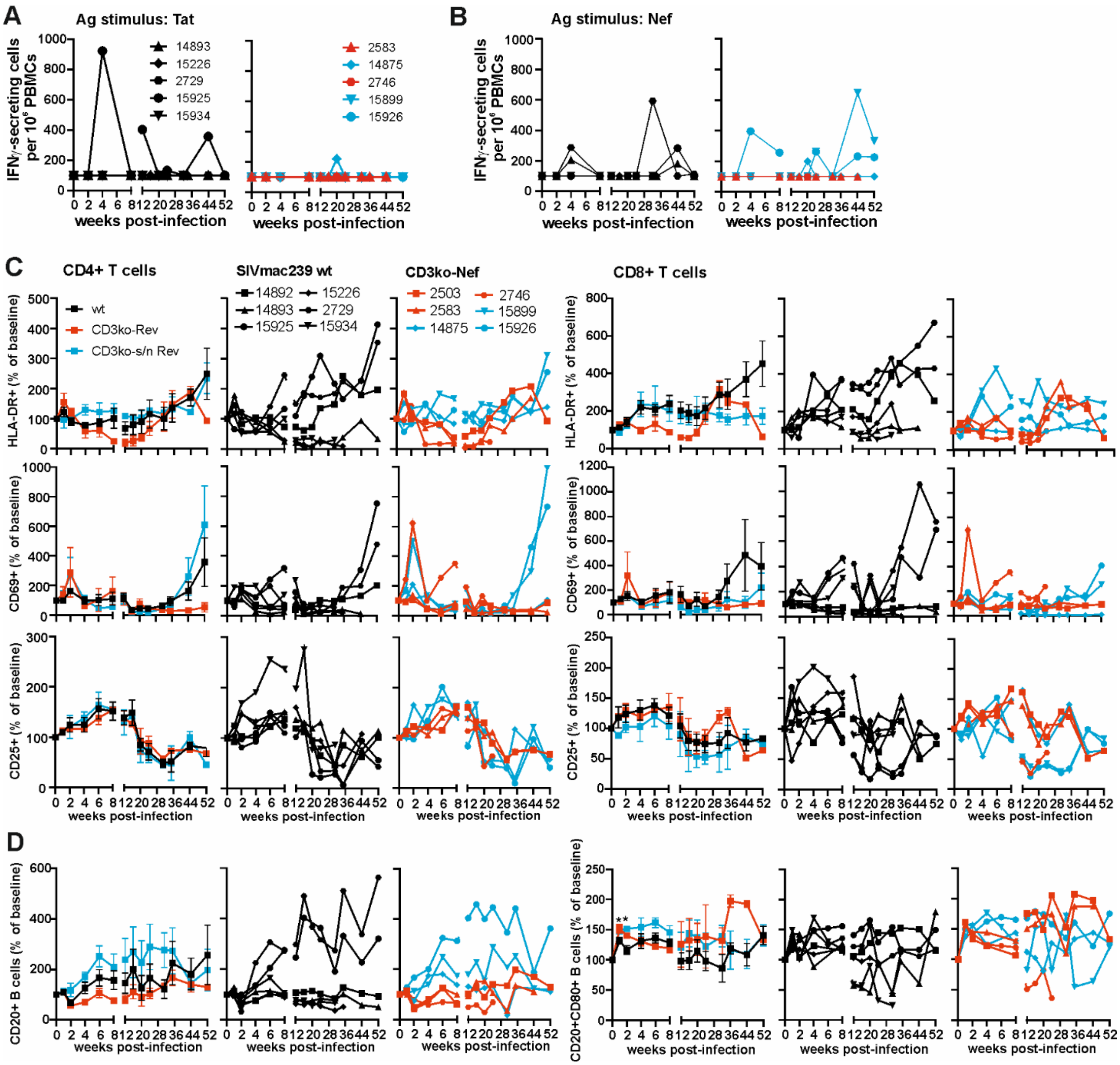

Figure S5 (related to Figure 5). Immunological findings during the first year of wt and CD3ko-Nef SIV$_{mac239}$ infection. (**A, B**) IFN-γ secreting lymphocytes as determined by ELISpot assay are shown as the number of spot-forming cells (SFC) per 10^6 PBMC after stimulation with (A) SIV-Tat or (B) SIV-Nef peptide pools. (**C**) Levels of activated HLA-DR+ (upper), CD69+ (middle) and CD25+ (bottom) CD4+ (left) and CD8+ (right) T cells in blood at the indicated weeks post-infection (wpi) relative to the baseline values (100%). The left panels show mean values (±SEM) measured for the wt (black) and CD3ko-Nef rapid (red) and no/slow (green) revertant groups and the middle and right panels values obtained for individual animals. Symbols specifying individual animals are indicated in the top left panel. (**D**) Percentages of total (left) and activated (CD80+, right) CD20+ B cells in blood at the indicated wpi relative to the baseline levels (100%).
